# Supplementary material for: Examining the predictive accuracy of metabolomics for small-for-gestational-age babies: a systematic review
Source: BMJ Open. 2019 Aug 10;9(8):e031238. doi: 10.1136/bmjopen-2019-031238 (PMC6701563; doi:10.1136/bmjopen-2019-031238)
Supplement: Supplementary data [file bmjopen-2019-031238supp001.pdf]

Examining the predictive accuracy of metabolomics for small for gestational age babies: a systematic review

Debora F. B. Leite and Aude-Claire Morillon; Elias F. de Melo Junior; Renato Teixeira Souza; Fergus P McCarthy; Ali S. Khashan; Philip N. Baker; Louise C. Kenny; Jose G. Cecatti

Supplementary material 1 – Detailed literature search strategy.

1	fetal growth retardation
2	fetal growth restriction
3	intrauterine growth restriction
4	intrauterine growth retardation
5	small for gestational age
6	#1 OR #2 OR #3 OR #4 OR #5
7	metabolomic*
8	metabonomic*
9	metabolit*
10	H NMR
11	proton NMR
12	proton nuclear magnetic resonance
13	liquid chromatogra*
14	gas chromatogra*
15	UPLC
16	ultra-performance liquid chromatograph*
17	ultra performance liquid chromatograph*
18	#7 OR #8 OR #9 OR #10 OR #11 OR #12 OR #13 OR #14 OR #15 OR #16 OR #17
19	pregnan*
20	antenat*
21	ante nat*
22	prenat*
23	pre nat*
24	#19 OR #20 OR #21 or #22 OR #23
25	screen*
26	predict*
27	metabolic profil*
28	#25 OR #26 OR #27
29	#6 AND #18 AND #24 AND 28
