# Supplementary material for: Examining the predictive accuracy of metabolomics for small-for-gestational-age babies: a systematic review
Source: BMJ Open. 2019 Aug 10;9(8):e031238. doi: 10.1136/bmjopen-2019-031238 (PMC6701563; doi:10.1136/bmjopen-2019-031238)
Supplement: Supplementary data [file bmjopen-2019-031238supp002.pdf]

Examining the predictive accuracy of metabolomics for small for gestational age babies: a systematic review

Debora F. B. Leite & Aude-Claire Morillon, Elias F. Melo Júnior, Renato T. Souza, Fergus P. McCarthy, Ali S. Khashan, Philip N. Baker, Louise C. Kenny, José Guilherme Cecatti.

Supplementary material 2 – List of excluded studies and reasons.

**Authors/ year**	**Country of enrollment**	**Additional comments**
Exclusions according to study design or statistical analysis		
Barnes CM et al, 2010	United States	Maternal samples collected at delivery.
Bobinski R. 2013	Poland	Cross-sectional study.
Bobinski R. 2014	Poland	Cross-sectional study.
Cao WC et al, 2016	China	Cross-sectional study. The metabolomics technique was not applied.
Chen TT et al, 2017	China	Cross-sectional study.
Cinelli et al, 2018	Italy	
D'Anna R et al, 2004	Italy	Cross-sectional study. The metabolomics technique was not applied.
Guo H et al, 2014	China	Cross-sectional study.

Guo J et al, 2016	China	Cross-sectional study.
Maekawa R et al, 2017	Japan	Cross-sectional study.
Mao D et al, 2010	China	Cross-sectional study.
Miranda J et al 2018	Spain	Cross-sectional study.
Powell et al, 2018	Australia	SGA babies not suspected before birth were considered healthy infants.
Spanou L. et al, 2017	Greece	Cross-sectional study.
Stein TP et al, 2008	United States	Newborns with birth defects were included in the analysis.
Tang R et al, 2013	China	Cross-sectional study.
Visentin S et al, 2017	Italy	Maternal samples collected after clinical recognition of FGR/SGA.
Zhu Y et al, 2018	China	Cross-sectional study.
Zota AR et al, 2009	United States	Cross-sectional study. The metabolomics technique was not applied.

Studies that have not applied metabolomics technique		
Baker PN, 2009	United Kingdom	
Berkowitz GS et al, 2004	United States	
Bodnar LM et al, 2012	United States	

Braun JM et al, 2011	United States	There is no data about FGR.
Cetin I et al, 2002	Italy	
Chong MFF et al, 2015	Singapore	There is no data about birth weight.
Colapinto CK et al, 2015	Canada	The metabolomics technique was not applied for pregnant women's specimens.
Cupul-Uicab LA et al, 2013	United States	
Fruscalzo A et al, 2015	Italy	There is no data about birth weight.
Jusko TA et al, 2006	United States	
Koepke R et al, 2004	Mexico	
Lõpez-Alarcõn M et al, 2015	Mexico	There is no data about birth weight.
Maruta E et al, 2017	Japan	
Miranda ML et al, 2015	United States	
Morley R et al, 2006	Australia	
Muthayya S et al, 2006	India	
Paşaoğlu H et al, 2003	Turkey	
Rahman A et al, 2009	Bangladesh	
Rajasingam D et al, 2009	United Kingdom	

Savitz DA et al, 2002	United States	The metabolomics technique was not applied for pregnant women's specimens.
Savvidou MD et al, 2003	United Kingdom	
Schneuer FJ et al, 2014	Australia	
Snijder CA et al, 2013	Netherlands	
Sweeney AM & Symanski E, 2007	United States	
Takimoto H et al, 2007	Japan	
Terrell ML et al, 2015	United States	
Wei Y et al, 2017	Bangladesh	
Weisskopf MG et al, 2005	United States	
Whyatt RM et al, 2009	United States	
Xue F et al, 2007	United States	

Studies that have not presented specific data about FGR/SGA		
Bach CC et al, 2016	Denmark	
Bachkangi P et al.	United Kingdom	
Bahado-Singh RO et al, 2012	United Kingdom	

Bahado-Singh RO et al, 2015	United Kingdom
Bahado-Singh RO et al, 2017	United Kingdom
Bentley-Lewis R, 2015	United States
Braun JM et al, 2009	United States
Buckley JP et al, 2016	United States
Cantonwine D et al, 2010	Mexico
Cantonwine D et al, 2015	United States
Casas M et al, 2016	Spain
Castorina R et al, 2017 (a)	United States
Chou WC et al, 2014.	Taiwan
Cunha Figueiredo AC et al, 2017	Brazil
Dalsager L et al, 2018	Denmark
De Renzy-Martin KT. et al, 2014	Poland
Debost-Legrand A et al, 2016	France
Desert et al, 2015	France

Diaz SO et al, 2011	Portugal
Diaz SO et al, 2013	Portugal
Dobierzewska A et al, 2017	Chile
Dudzik D et al, 2015	Spain.
Engström KS et al, 2010	Bangladesh
Ettinger AS et al, 2017	Canada
Feng L et al, 2016	China
Ferguson KK et al, 2014	United States
Ferguson KK et al, 2015	United States
Ferguson KK et al, 2017	United States
Finkelstein JL et al, 2015	United States
Fischer ST et al, 2017	United States
Gao H et al, 2017	China
Gardner RM et al, 2011	Bangladesh
Ghartey J et al, 2017	United States
Graça G et al, 2010	Portugal

Graça G et al, 2012	Portugal	
Graça G et al, 2012 (b)	Portugal	
Hogeveen M et al, 2010	Netherlands	
Huang J et al, 2017	China	
Kalhan SC et al, 2003	United States	
Khalil AA et al, 2013	United Kingdom	
Kuc S et al, 2014	Netherlands	
Lenters V et al, 2013	Greenland, Poland, Ukraine	
Lenters V et al, 2016	Greenland, Poland, Ukraine	
Liu K et al, 2017	China	
Lopez-Espinosa MJ et al, 2015	Spain	
Marchlewicz EH et al, 2016	United States	
Minatoya M et al, 2017	Japan	
Minatoya M et al, 2017 (b)	Japan	
Minatoya M et al, 2018	Japan	
Murphy MM et al, 2007	Spain	There is no data about any pregnancy outcomes.

Odibo AO et al, 2011	United States	
Pinney SE et al, 2017	United States	
Polanska K et al, 2014	Poland	
Polanska K et al, 2014 (b)	Poland	
Porter A et al, 2018	United States	
Rejc B et al, 2016	Slovenia	
Rijvers CAH et al, 2013	Netherlands	
Robledo C et al, 2013	United States	
Sachse D et al, 2012	Norway	
Scholtens DM et al, 2016	United Kingdom	
Shisler S et al, 2017	United States	Not all analysis were performed with metabolomics approach.
Tamblyn JA et al, 2018	Ireland	Duplicate data. Check Kiely ME et al, 2016.
Thomas MM et al, 2015	New Zealand	
Van Lee L et al, 2015	Singapore	
Virgiliou C et al, 2017	Greece	
Walsh J et al, 2012	Ireland	

Wang PW et al, 2015	Taiwan	
Watkins DJ et al, 2016	United States	
Wolff MS et al, 2008	United States	
Woods MM et al, 2017	United States	
Yang P et al, 2018	China	

Duplicate data		
Horgan R et al, 2009	Australia	Check Horgan R et al, 2011.
Horgan R et al, 2011	Australia	Check Horgan R et al, 2011.
Khashan AS et al, 2013	Ireland	Check Kiely ME et al, 2016.
Sulek et al, 2014	Singapore	Check Sulek et al, 2014.
