# Supplementary material for: Examining the predictive accuracy of metabolomics for small-for-gestational-age babies: a systematic review
Source: BMJ Open. 2019 Aug 10;9(8):e031238. doi: 10.1136/bmjopen-2019-031238 (PMC6701563; doi:10.1136/bmjopen-2019-031238)
Supplement: Supplementary data [file bmjopen-2019-031238supp003.pdf]

Examining the predictive accuracy of metabolomics for small for gestational age babies: a systematic review

Debora F. B. Leite & Aude-Claire Morillon, Elias F. Melo Júnior, Renato T. Souza, Fergus P. McCarthy, Ali S. Khashan, Philip N. Baker, Louise C. Kenny, José Guilherme Cecatti.

Supplementary material 3 - Individual QUADAS-2 data for all 15 included studies.

Studies	Risk of bias							
	**Patient selection**		**Index test**		**Reference standard**		**Flow and timing**	
	Was a consecutive or random sample of patients enrolled?	Did the study avoid inappropriate exclusions?	Were the index test results interpreted without knowledge of the results of the reference standard?	If a threshold was used, was it pre-specified?	Is the reference standard likely to correctly classify the target condition?	Were the reference standard results interpreted without knowledge of the results of the index test?	Did all patients receive the same reference standard?	Were all patients included in the analysis?
Grandone E et al, 2006	Yes	Yes	Unclear	No	No	Unclear	Yes	Yes
van Eijsden M et al, 2008	Yes	Yes	No	No	Yes	Yes	Yes	Yes
Horgan R et al, 2011	Yes	Yes	No	Yes	Yes	Yes	Yes	Yes
Costet N et al, 2012	Yes	Yes	Yes	No	Yes	Unclear	Yes	No
Ertl R et al, 2012	Yes	Yes	No	Yes	Yes	Yes	Yes	Yes
Gernand AD et al, 2013	Yes	Yes	No	Yes	Yes	Yes	Yes	Yes
Sulek K et al, 2014	Yes	Yes	No	Yes	Unclear	Unclear	Yes	Yes
Choi R et al, 2016	Yes	Yes	Unclear	Yes	Yes	Unclear	Yes	Yes
Kiely ME et al, 2016	Yes	Yes	No	Yes	Yes	Yes	Yes	Yes
Ong YL et al, 2016	Yes	Yes	No	Yes	Yes	Yes	Yes	No
Wang Y et al, 2016	Yes	Yes	No	No	Yes	Yes	Yes	Yes
Delplancke TDJ et al, 2018	Yes	Yes	No	Yes	Yes	Unclear	Yes	Yes
Luthra G et al, 2018	Yes	Yes	No	No	Yes	Yes	Yes	Yes
Gong S et al, 2018	No	Yes	No	No	Yes	Unclear	Yes	Yes
Morillon AC et al, 2018	Yes	Yes	No	Yes	Yes	Yes	Yes	Yes

Studies	Applicability concerns		
	**Patient selection**	**Index test**	**Reference standard**
	Are there concerns that the included patients do not match the review question?	Are there concerns that the index test, its conduct, or interpretation differ from the review question?	Are there concerns that the target condition as defined by the reference standard does not match the review question?
Grandone E et al, 2006	No	No	Yes
van Eijsden M et al, 2008	No	No	No
Horgan R et al, 2011	No	No	No
Costet N et al, 2012	No	No	No
Ertl R et al, 2012	No	No	No
Gernand AD et al, 2013	No	No	No
Sulek K et al, 2014	No	No	Yes
Choi R et al, 2016	Unclear	No	No
Kiely ME et al, 2016	No	No	No
Ong YL et al, 2016	No	No	No
Wang Y et al, 2016	No	No	No
Delplancke TDJ et al, 2018	No	Unclear	No
Luthra G et al, 2018	No	No	No
Gong S et al, 2018	Yes	Yes	No
Morillon AC et al, 2018	No	No	No
