# Supplementary material for: Examining the predictive accuracy of metabolomics for small-for-gestational-age babies: a systematic review
Source: BMJ Open. 2019 Aug 10;9(8):e031238. doi: 10.1136/bmjopen-2019-031238 (PMC6701563; doi:10.1136/bmjopen-2019-031238)
Supplement: Author's manuscript [file bmjopen-2019-031238.draft_revisions.pdf]

BMJ Open

BMJ Open is committed to open peer review. As part of this commitment we make the peer review history of every article we publish publicly available.

When an article is published we post the peer reviewers' comments and the authors' responses online. We also post the versions of the paper that were used during peer review. These are the versions that the peer review comments apply to.

The versions of the paper that follow are the versions that were submitted during the peer review process. They are not the versions of record or the final published versions. They should not be cited or distributed as the published version of this manuscript.

BMJ Open is an open access journal and the full, final, typeset and author-corrected version of record of the manuscript is available on our site with no access controls, subscription charges or pay-per-view fees (http://bmjopen.bmj.com).

If you have any questions on BMJ Open's open peer review process please email info.bmjopen@bmj.com

BMJ Open

Examining the predictive accuracy of metabolomics for small for gestational age babies: a systematic review

Journal:	*BMJ Open*
Manuscript ID	bmjopen-2019-031238
Article Type:	Research
Date Submitted by the Author:	23-Apr-2019
Complete List of Authors:	Leite, Debora; Campinas' State University, Department of Tocogynecology; Universidade Federal de Pernambuco,<br>Morillon, Aude-Claire; University College Cork National University of Ireland, Irish Centre for Fetal and Neonatal Translational Research (INFANT)<br>Melo Júnior, Elias; Universidade Federal de Pernambuco, Clinics Hospital<br>Souza, Renato; Universidade Estadual de Campinas, Obstetrics and Gynecology;<br>McCarthy, F. P.; St Thomas Hosp<br>Khashan, Ali; University College Cork, Department of Epidemiology and Public Health<br>Baker, Philip ; University of Leicester, College of Medicine<br>Kenny, Louise; University of Liverpool School of Life Sciences, Department of Women's and Children's Health<br>Cecatti, Jose; University of Campinas, Obstetrics and Gynecology
Keywords:	small for gestational age, fetal growth restriction, metabolomics, gas-chromatography, mass spectrometry

SCHOLARONE™
Manuscripts

RESEARCH ARTICLE: SYSTEMATIC REVIEW

Examining the predictive accuracy of metabolomics for small for gestational age babies: a systematic review

Debora F. B. LEITE[a,b,c1] (0000-0001-8839-3934; deborafariasleite@gmail.com) and Aude-Claire MORILLON[d1] (0000-0002-9692-9396; audeclaire.morillon@ucc.ie); Elias F. de MELO JUNIOR[a,b] (0000-0001-7897-8090; eliasmelojr@gmail.com); Renato Teixeira SOUZA[c] (0000-000209075-9269; renatotsouzasp@gmail.com); Fergus P McCARTHY[d] (0000-0001-5062-6851; fergus.mccarthy@ucc.ie); Ali S. KHASHAN[d,e] (0000-0002-7499-9406; a.khashan@ucc.ie); Philip N. BAKER[f] (0000-0002-4592-6427; philip.baker@leicester.ac.uk); Louise C. KENNY[g] (0000-0002-9011-759X; louise.kenny@liverpool.ac.uk); Jose G. CECATTI[c] (0000-0003-1285-8445; cecatti@unicamp.br).

[a]Federal University of Pernambuco, Pernambuco, Brazil.

[b]Clinics Hospital of Federal University of Pernambuco, Recife, Pernambuco, Brazil

[c]Department of Gynecology and Obstetrics, University of Campinas, Campinas, Sao Paulo, Brazil.

[d]The Irish Centre for Fetal and Neonatal Translational Research (INFANT), University College Cork, Cork, Co. Cork, Ireland.

[e]School of Public Health, University College Cork, Cork, Co. Cork, Ireland.

[f]College of Life Sciences, University of Leicester, United Kingdom.

[g]Faculty of Health and Life Sciences, Department of Women's and Children's Health, Institute of Translational Medicine, University of Liverpool, United Kingdom.

For peer review only

[1]These two authors contributed equally.

CORRESPONDING AUTHOR

José Guilherme Cecatti

Rua Alexander Fleming, 101 Cidade Universitaria Zeferino Vaz.

13083-889. Campinas, Sao Paulo, Brazil.

Telephone: +55 19-35219482

Email: cecatti@unicamp.br

Word Count:

Abstract: 296 words

Main text 4,106 words

For peer review only

ABSTRACT

Introduction: To date, there is no robust enough test to predict small for gestational age (SGA) infants, which are at increased life-long risk of morbidity and mortality.

Objective: To determine the accuracy of metabolomics in predicting SGA babies and elucidate which metabolites were found to be predictive of this condition.

Data sources: Two independent researchers explored 11 electronic databases and grey literature in February 2018 and November 2018, covering publications from 1998 to 2018. Both researchers performed data extraction and quality assessment independently. Discrepancies were resolved by a third researcher.

Study eligibility criteria: Cohort or nested case-control studies were included, which investigated pregnant women and performed metabolomics analysis to evaluate SGA infants. The primary outcome was birthweight <10th centile - as a surrogate for fetal growth restriction - by population-based or customized charts.

Study appraisal and synthesis methods: Data on study design, obstetric variables and sampling, metabolomics technique, chemical class of metabolites, and prediction accuracy measures were extracted by two independent researchers. Authors were contacted to provide additional data when necessary.

Results: A total of 9,181 references were retrieved. Of these, 273 were duplicate, 8,760 were removed by title or abstract, and 133 were excluded by full text content. Thus, 15 studies were included. Only two studies used the 5th centile as a cutoff, and most reports sampled 2nd trimester pregnant women. Liquid-chromatography coupled to mass spectrometry was the most common metabolomics approach. Untargeted studies in the 2nd trimester provided the largest number of predictive metabolites, using maternal blood or hair. Fatty acids, phosphosphingolipids, and amino acids were the most prevalent predictive chemical subclasses.

For peer review only

Conclusions and Implications: Significant heterogeneity of participant characteristics and methods employed among studies precluded a meta-analysis. Compounds related to lipid metabolism should be validated up to the 2nd trimester in different settings.

Systematic review registration number: CRD42018089985.

Keywords: small for gestational age, fetal growth restriction, metabolomics, prediction, gas-chromatography, mass spectrometry, vitamin D, homocysteine, lipids, fatty acids.

STRENGHTS AND LIMITATIONS OF THIS STUDY

- To our knowledge, this is the first systematic review to assess the predictive accuracy of metabolomics for an adverse pregnancy outcome.
- Using SGA as surrogate for fetal growth restriction – just as in epidemiological investigations – improves the translational potential of metabolomics.
- Identification of techniques, types of maternal samples and chemical classes paves the way for future metabolomics investigations on fetal growth patterns.
- Available data could not support a meta-analysis; further studies should include accuracy measures of individual metabolites or chemical subclasses in predicting SGA.

ORIGINAL PROTOCOL: Leite DFB, Morillon A-C, Melo Júnior EF, *et al*. Metabolomics for predicting fetal growth restriction: protocol for a systematic review and meta-analysis. *BMJ Open* 2018;8:e022743. doi:10.1136/bmjopen-2018-022743.

For peer review only

INTRODUCTION

Fetal growth restriction (FGR) and small for gestational age (SGA) infants are major concerns in modern obstetrics. [1–3] SGA is commonly used as a proxy for FGR, [4] despite the subtle differences between these two pathological conditions. The prevalence of both varies according to criteria applied and on the population and setting, although it reaches as much as 25% in low and middle-income countries. [5] SGA newborns may have adverse health effects, such as stillbirth, [4] perinatal asphyxia, [6] impaired neurodevelopment, [7] and increased cardiovascular risk. [8,9] To date, there are no robust prediction tools for SGA using clinical factors, [10,11] ultrasound data, [12,13] or placental biomarkers. [14]

For hypothesis generating or validation purposes, metabolomics is a novel area of biomarker, discovery, development and clinical diagnostics in translational medicine. [15,16] Metabolomics is the study of all metabolites [15,16] in a given sample, i.e. low molecular weight compounds (50-2000 Da) that are intermediates of biochemical reactions and metabolic pathways, considered to directly reflect cellular activity and phenotype. [15,16] Recent studies have evaluated the pathophysiology [17–20] of SGA with metabolomics. However, little is known about the potential of metabolomics to identify predictive compounds of SGA.

Since metabolomics can identify multiple metabolites from low volume samples, and create a model from a collection of these samples, [15] it is a promising technology for hypothesis generation in a heterogeneous condition such as SGA. The prediction of SGA in pregnancy would help refer women to specialized care facilities, improving maternal and neonatal outcomes. [21,22]

For peer review only

In this context, the main objective of this systematic review was to assess the accuracy of metabolomics techniques in predicting SGA. As a secondary aim, we intended to determine which metabolites are predictive of this condition.

METHODS

The protocol for this systematic review was published previously. [23] This study follows international guidelines for transparency (PROSPERO, CRD 42018089985) and respects the Preferred Reporting Items for Systematic Reviews and Meta-Analysis (PRISMA) statement. [24] This systematic review was conducted without any public involvement, and ethical approval was unnecessary.

Literature Search Strategy

Two independent researchers (DFBL and ACM) assessed 11 electronic databases (PubMed, EMBASE, Latin American and Caribbean Health Sciences Literature (LILACS), Scientific Electronic Library Online (Scielo), Health Technology Assessment (HTA), Database of Abstracts of Reviews of Effects (DARE), Aggressive Research Intelligence Facility (ARIF), Cumulative Index of Nursing and Allied Health Literature (CINAHL), Maternity and Infant Care (MIDIRS), Scopus, and Web of Science) and grey literature. There were no limits or language constraints; the search strategy covered published documents between 1998 and 2018. Keywords 'small for gestational age', 'metabolomics', 'prediction', 'antenatal', and variations of each, were combined with Boolean operators depending on each database requirements. The full EMBASE literature search was, as follows: ('fetal growth retardation' OR 'fetal growth restriction' OR 'intrauterine growth restriction' OR 'intrauterine growth retardation' OR 'small for gestational age') AND ('metabolomic*' OR 'metabonomic*'

For peer review only

OR ‘metabolit* ‘H NMR’ OR ‘proton NMR’ OR ‘proton nuclear magnetic resonance’ OR ‘liquid chromatogra*’ OR ‘gas chromatogra*’ OR ‘UPLC’ OR ‘ultra-performance’ OR ‘ultra performance liquid chromatograph*’) AND (‘pregnan*’ OR ‘antenat*’ OR ‘ante nat*’ OR ‘prenat*’ OR ‘pre nat*’) AND (‘screen*’ OR ‘predict*’ OR ‘metabolic profil*’).

Outcomes and subgroup analysis

The primary outcome was SGA, as a surrogate for FGR and defined as birthweight <10th centile, by population-based or customized charts. Secondary outcomes were birthweight ≤5th or ≤3rd centile.

The intended subgroup analysis comprised: type of metabolomics technique applied (nuclear magnetic resonance, NMR; gas or liquid chromatography coupled with mass spectrometry, GC-MS or LC-MS respectively); maternal health status before pregnancy (women with *versus* without any chronic health condition); type of SGA suspected during pregnancy (early *versus* late SGA); and type of pregnancy (singleton *versus* multiple pregnancy).

Selection Criteria of Studies, Data Collection and Analysis

Cohort or case-control studies were included if maternal samples were collected before the clinical diagnosis of SGA, if any metabolomics technique was applied, and if the results of SGA were presented. Articles presenting data from the same research project but analyzing distinct metabolites or showing data from different countries were included. Studies were excluded (i) according to study design; (ii) if they had not applied any metabolomics technique; (iii) if they were only experimental studies; (iv) if it was not possible to extract data on SGA; (v) or if they presented

For peer review only

duplicate data, in which case the most complete publication was included for final analysis.

Two researchers (DFBL and ACM) independently selected studies, extracted data and discussed discrepancies. One additional reviewer (EFMJ or RTS) helped to decide, by majority, when no consensus was reached.

Piloted standardized forms were applied for data extraction, including pregnancy characteristics and experimental details. The Human Metabolome Database (HMDB) [25] and the Kyoto Encyclopedia of Genes and Genomes [26] were used for matching chemical class and metabolic pathways of each metabolite, respectively.

Risk of bias and Assessment of concerns regarding applicability

Two researchers (DFBL and ACM) independently evaluated individual studies using the QUADAS-2 tool. [27] One of the third reviewers (EFMJ, or RTS) helped in decision-making when no consensus was achieved.

Each study was classified as high, low, or unclear risk of bias in four Domains (Patient Selection, Index Test, Reference Standard, and Flow and Timing), and as high, low, or unclear concerns regarding applicability in the first three Domains. We did not consider two signaling questions (“Was a case-control design avoided?”, “Was there an appropriate interval between the index test and reference standard?”). The nested case-control design was an inclusion criterion and maternal samples should have been collected during pregnancy, i.e. before the SGA diagnosis. Studies were considered ‘low risk’, for example, (i) if pregnancy or neonatal complications were not excluded in just one group of participants or data on participant selection had been provided; (ii) if methods for sample preparation and

For peer review only - http://bmjopen.bmj.com/site/about/guidelines.xhtml

interpretation were standardized or metabolite threshold was defined before the experiments (for targeted analysis); (iii) if the adequacy and reasons for choosing the reference birthweight chart had been explained; or, (iv) if large for gestational age babies had been excluded from the final comparative analysis.

Data synthesis

A quantitative summary of data was performed when any predictive accuracy measures could be extracted. Authors were contacted to provide additional information, when necessary. However, only Delplancke et al [28] replied. The estimation of likelihood ratios and hierarchical summary receiver operator characteristic curve [29] were planned, as well as assessment of heterogeneity and publication bias. [30] However, due to lack of data, a meta-analysis could not be performed.

RESULTS

Literature search characteristics

The literature search for this systematic review was performed in February 2018, and re-run in November 2018. A total of 9,181 references were retrieved (Figure 1). After the removal of duplicate records (n=273), title and abstract screening, and analysis of the remaining 148 full-text articles, 15 articles were included. [17,18,38–42,28,31–37] See Supplementary Material 1 for excluded studies.

Characteristics of the included studies

The characteristics of the included studies are shown in Table 1. The prevalence of SGA ranged from 7.3% [33] to 21.5% in cohort studies. [28] There were no studies

For peer review only

using a birthweight ≤3rd centile for a definition of SGA. The time interval between initial participant enrollment and publication varied from three [17] to 54 years, [40] although these data were unclear in 38% of the reports. [18,28,32,33,37] In nested case-control studies, participants were matched by maternal age, [17,18,38,42] ethnicity, [17,18,42] parity, [38] body mass index, [17,18,42] or infant gender. [18,38]

Participant characteristics varied between studies. Regarding gestational age at assessment, samples were collected in the 2nd trimester in one half of the studies. [17,18,33,35,37,39,42] In three reports, women were assessed at least twice. [34,38,41] In one study, maternal blood was drawn either in the 1st or 2nd trimester; [40] and in another three studies, only samples from the 3rd trimester were considered. [28,36,41] In the latter case, maternal hair was divided according to length, allowing evaluation of 2nd and 3rd trimester metabolites. [28] Studies considering the 5th centile as the cutoff, sampled women in the 1st trimester. [31,32] Twin pregnancy was a clear exclusion criterion in most studies. [17,18,31,33–35,37,40–42] Pregnancy aided by assisted reproduction [18,37] or women with pre-existing conditions [17,18,35,37,42] were also excluded, although these data were incompletely reported. [28,32,36,38,39,41] When both nulliparous and multiparous women were enrolled, there was no data analysis according to parity. Half of the studies considered term deliveries exclusively, [18,28,36,38–41] and the remaining studies did not differentiate results according to gestational age at birth.

Regarding clinical risk factors for SGA, only one paper mentioned a previous history of SGA, but findings were not adjusted for this variable. [32] All studies, except one, [28] cited participant smoking status. The rate of smoking habit ranged from 2.4% [18] to 47.5%. [40] It is important to note that Gernand et al [40] analyzed samples from women recruited between 1959 and 1965, when smoking while

For peer review only

pregnant was encouraged, which explains the high rate of smoking participants. The duration of smoking or any differences in birthweight (absolute measures or centiles) were not clearly stated. Although more prevalent in SGA pregnancies, results did not change with this variable control. [31,32,35,37,40] Only Gong et al [41] mentioned the suspicion of SGA in pregnancy, exhibiting decreasing abdominal circumference growth velocity between 20-36 wks. However, on final analysis, these babies were grouped with infants not suspected during pregnancy.

For peer review only

Table 1. Main characteristics of included studies

Authors, year	Country, year of participants enrolment	Study design	Affected/ non-affected	Gestational age at assessment	Type of pregnancy	Parity	Birthweight curve
**Outcome: SGA <5th centile**							
Costet N et al, 2011	France, 2002-2006 (PELAGIE Cohort)	Nested case-control	134/ 399	11w	Single pregnancy	Nulliparous and parous women, unclear proportions	Customized curve
Ertl R et al, 2012	United Kingdom [a]	Nested case-control	150/ 1,000	11^{+0}-13^{+6}w	Unclear	55,3% nulliparous in SGA group, 48.1% nulliparous in control group	Population-based charts
**Outcome: SGA <10th centile**							
Grandone E et al, 2006	Italy [a]	Cohort	31/ 393	17.1 ± 1.2w[b] (mean)	Single pregnancy; no maternal pre-existing conditions	Unclear	Population-based charts
van Eijsden M et al, 2008	Netherlands, 2003-2004 (ABCD Study)	Cohort	429/ 3275	13.5 ± 3.3w (mean)	Term deliveries, no diabetes or hypertension	57.6% nulliparous	Population-based charts
Horgan RP et al, 2011	Australia, 2008-2011 (SCOPE Cohort)	Nested case-control	40/ 40	14-16w	Single pregnancy; no other pregnancy complications	Nulliparous	Customized curve
Gernand AD et al, 2013	United States, 1959-1965 (Collaborative Perinatal Project)	Nested case-control	395/ 1751	≤26w	Single pregnancy; term deliveries	Parous women	Population-based charts
Sulek K et al, 2014	Singapore [a] (GUSTO Study)	Nested case-control	41/ 42	26-28w	Single pregnancy; term deliveries; no maternal pre-existing conditions	Nulliparous and parous women, unclear proportions	Population-based charts
Choi R et al, 2016	South Korea, 2012-2013	Cohort	39/ 217	1st, 2nd or 3rd trimester	Single pregnancies	Nulliparous and parous women, unclear	Population-based charts

For peer review only

						proportions	
Kiely ME et al, 2016	Ireland, 2008-2011 (SCOPE Cohort)	Cohort	190/ 1578	14-16w	Single pregnancy; no maternal pre-existing conditions	Nulliparous	Customized curve
Ong YL et al, 2016	Singapore [a] (GUSTO Study)	Cohort	83/ 827	26-28w	Single pregnancy; no maternal chronic illness	43,5% nulliparous	Population-based charts
Wang Y et al, 2016	Taiwan, 2000-2001 (Taiwan Maternal and Infant Cohort Study)	Cohort	35/ 188	3rd trimester	Unclear; term deliveries	48% nulliparous	Population-based charts
Delplancke TDJ et al, 2018	New Zealand [a]	Cohort	20/ 73	34-37w	Unclear; term deliveries	Unclear	Customized curve
Luthra G et al, 2018	United States, 2010-2012 (TIDES Study)	Nested case-control	53/ 106	1st and 2nd trimester	Single pregnancies; term deliveries	60% nulliparous	Customized curve
Gong S et al, 2018	United Kingdom, 2008-2012 (POP study)	Nested case-control	162/259	36w	Single pregnancies; term deliveries	Nulliparous	Customized curve
Morillon A-C et al, 2018	2008-2011 (SCOPE Study)	Nested case-control	40/40	20w	Single pregnancies	Nulliparous	Customized curve

[a] Unclear period of participant recruitment. [b] Mean for all study participants.

For peer review only

Subgroup analysis

Due to unavailable data, the only subgroup analysis performed was related to the metabolomics approach applied (Table 2). There was no mention of adherence to metabolomics reporting data guidelines. LC-MS was the leading technique used. Three studies have investigated metabolites related to environmental exposure, from contaminated water, [31] consumer products,[36] or pesticides, [42] while others have analyzed endogenous compounds. [32–35,37–40] Only Luthra et al conducted a biomarker validation study, [38] while Gong et al [41] chose to analyze the top ten statistically different metabolites according to infant sex.

Maternal blood was the most common biological sample analyzed by LC-MS in all studies, [17,32,34–37,39–41] except for one, which used GC-MS.[39] Maternal urine was analyzed by NMR, [38] GC-MS [36] or LC-MS. [42] There was only one report using amniotic fluid [33] and two using maternal hair, [18,28] all applying GC-MS. The period of laboratory analysis was rarely specified, which made it impossible to estimate total time of sample storage.

Untargeted studies reported diverse metabolic features. Authors matched the peaks with an in-house library [18,28] or HMDB-related database. [17,42] Horgan et al [17] found 785 compounds both in maternal and newborn samples; their predictive model included 19 metabolites (only five could be putatively identified, Table 2) and used 2nd trimester maternal blood. Sulek et al [18] and Delplancke et al [28] prepared and analyzed samples with GC-MS using similar protocols. Sulek et al [18] identified 32 statistically different chromatographic features from which they built a predictive model using five metabolites, including two fatty acids (2-methyloctadecanoate and margarate). In contrast, Delplancke et al, [28] identified 198 metabolites, including three fatty acids (margaric, pentadecanoic, and myristic

For peer review only

acid) showing significantly higher levels in SGA cases, when 2nd trimester maternal hair segments were studied.

For peer review only

Table 2. Subgroup analysis of included studies according to which metabolomics technique was applied.

Authors/ year	Metabolomics Technique	Maternal sample/ Storage temperature	Prediction model*	Targeted compounds	Coefficient of variation/ Limits of quantitation	Predictive compounds	Sensitivity/ Specificity	AUC
**Nuclear magnetic resonance**								
Luthra G et al, 2018	^{1}H-NMR 1D NOESY with pre-saturation and homonuclear 2D *J*-resolved at 300 K<br>Bruker 600 MHz Advance III HD spectrometer	Urine/ -80°C	Targeted	Tyrosine, acetate, formate, trimethylamine	NA	None		
**Gas chromatography coupled to mass spectrometry**								
Costet N et al, 2011	GC-MS<br>Simple head space SPME-Capillary GC	Urine/ -20°C	Targeted	Trichloroacetic acid	<5%/ 0.01mg/L	None	0.1/ 0.93	
Sulek K et al, 2014	GC-MS<br>Thermo Trace GC Ultra system coupled to ISQ mass selective detector<br>Capillary GC column: Phenomenex ZB-1701 (30 m x 250 µm id x 0.15 µm with 5 m guard column)	Hair/ -20°C	Untargeted	NA	NA	↓ Lactate<br>↓ Levulinate<br>↑2-methyloctadecanate<br>↑Tyrosine<br>↓ Margarate		0.998
Delplancke TDJ et al, 2018	GC-MS:<br>Agilent 7890B gas chromatograph, capillary column ZB-1701 (30m x 250µm id x 0.15µm with 5m guard column)<br>5977 A mass spectrometer, electron impact ionisation	Hair/ -20°C	Untargeted	NA	NA	↑ Margaric acid<br>↑ Pentadecanoic acid<br>↑ Myristic acid[c]		0.72<br>0.73<br>0.73

**Liquid chromatography coupled to mass spectrometry**								
Grandone E et al, 2006	LC-MS/MS triple quadrupole Applera API 3000, TurboIonSpray ionisation	Amniotic fluid/ -80^oC	Targeted	Homocysteine	Unclear	↑Homocysteine (1,29µM; 1,05-1,51µM)		
Horgan RP et al, 2011	UPLC- MS/MS Thermo Fisher LTQ Orbitrap, ESI	Plasma/ -80^oC	Untargeted	NA	NA	Hexacosanedioic acid, diglyceride, lyso-phosphocholine, sphinganine 1-phosphate; sphingosine 1-phosphate[d]		0.90
Ertl R et al, 2012	HPLC- MS/MS Shimadzu Prominence HPLC system with a column Phenomenex Luna C8 3 x 50 mm; AbSciex API-5000 triple quadrupole, ESI	Serum/ -80^oC	Targeted	25(OH)D_2; 25(OH)D_3	6.3%[a], 6.6%[b] (D_2); 6.5%[a], 7.3%[b] (D_3)/ unclear	↓25,OH,Vitamin D (12.16ng/mL; 8.09-20.54ng/mL)	0.72/ 0.45	
Gernand AD et al, 2013	LC-MS/MS	Serum/ -20^oC	Targeted	25(OH)D_2; 25(OH)D_3	8.2%[a] (D_2) 5.9%[a] (D_3)/ <1ng/mL	None	0.39/ 0.66	
Choi R et al, 2016	HPLC- MS/MS Waters HPLC system, Applied Biosystems API-4000 MS/MS mass spectrometer	Serum/ -20^oC	Targeted	Methylmalonic acid; homocysteine	<10%[a]; <10%[b]/ Unclear	None		
Kiely ME et al, 2016	UPLC- MS/MS Waters Acquity UPLS system, Waters Triple Quadrupole TQD mass spectrometer	Serum/ -80^oC	Targeted	25(OH)D_2; 25(OH)D_3; 3-epi-25(OH)D_3.	<6%[a]; <5%[b]/ 0.57ng/mL (D_2); 0.26ng/mL (D_3), 0,41ng/mL (epi-D_3)	None		

Ong YL et al, 2016	LC-MS/MS Applied Biosystems ThermoHypersil BDS C8 reverse-phase column	Plasma/ Unclear	Targeted	25(OH)D_2; 25(OH)D_3	≤10,3%[a,b]/ <1,6ng/mL	None	0.12/ 0.87
Wang Y et al, 2016	LC-MS Agilent HPLC system, Applied Biosystems Sciex API-4000 triple quadrupole mass spectrometer	Serum/ Unclear	Targeted	PFOA; long-chain PFCA	0,83-7,94%[a]; 1,57-24,7%[b]/ 0,07-0,45ng/mL[e]	PFDeA (OR 3,14; 95%CI 1,07-9,19), PFUnDA (OR 1,83; 95%CI 1,01-3,32)[f]	
Gong S et al, 2018	LC-MS/MS Shimadzu UK Limited UPLC system, ACE Excel 2 C18-PFP LC-column; Thermo Fisher Scientific Exactive orbitrap mass spectrometer	Serum/ Unclear	Untargeted	NA		↑N1,N12-diacetylspermine[f]	
Morillon A-C et al, 2018	UPLC- MS/MS Waters Acquity UPLS system, Waters Synapt G2-S mass spectrometer	Urine/-80oC	Untargeted	NA		None	
Others							
van Eijsden M et al, 2008	GC-FID Solid phase extraction SPE, Capillary GC	Plasma/ -80°C	Semi-targeted, Lipid extraction	Elaidic, linoleic, alfa-linolenic, eicosatetraenoic, EPA, DPA, DHA DGLA, AA, Adrenic, and Osbond acids	≤2 - 22%[b]/ Unclear	↓ Eicosatetraenoic acid (OR 1,5; 95%CI 1,07-2,11), ↓DPA (OR 1,49; 95% CI 1,06-2,1)	

[a]Intra-assay and [b]inter-assay coefficients of variation. [c]These metabolites were found in 2nd trimester hair segments. [d]And more 14 metabolites that could not be identified certain based on chromatographic peak and mass: Phenylacetylglutamine or formyl-N-acetyl-5-methroxykynurenamine; leucyl-leucyl-norleucine or sphingosine 1-phosphate; cervonyl carnitine and/or 1-alpha,25-dihydroxy-18-oxocholecalciferol; (15Z)-tetracosenoic acid or 10,13-dimethyl-11-docosyne-10,13-diol or trans-selacholeic acid; pencosenoic acid or cyclohexyl acetate or octanoic acid or methyl-heptenoic acid or 4-hydroxy-2-octenal or DL-2-aminooctanoic acid or 3-amino-octanoic acid; hydroxybutyrate or hydroxy-methylpropanoate or methyl methoxyacetate; lysophosphocoline and phosphocoline (more than 10 hits); phosphocoline (more than 20 hits); phosphocoline or ubiquinone-8; acetylleucil-leucil-norleucinal or oleoylglycerone phosphate or LPA(0:0/18:2(9Z,12Z)) or 1-16:1lysoPE or phosphocoline(O-11:1(10E)/2:0) or (3s)-3,4-Di-N-hexanoyloxybutyl-1-phosphocoline or N-(3-hydroxy-propyl) arachidonoyl amine or N-methyl N-(2-hydroxy-ethyl) arachidonoyl amine or similar; lysophosphocholine (16:1) or cervonyl carnitine; preganediol-3-glucuronide or 3-alpha,20-alpha-dihydroxy-5-beta-pregnane-3-glucuronide; 6-hydroxyshingosine or

For peer review only

For peer review only - http://bmjopen.bmj.com/site/about/guidelines.xhtml

(4OH,8Z,t18:1) sphingosine or 15-methyl-15-prostaglandin D2 or 15-R-prostaglandin E2 methylester. [e]Values for all studied metabolites. [f]Predictive compounds only for female babies.

AUC: area under the receiver operating characteristic curve; ^1H-NMR: hydrogen nuclear magnetic resonance; NOESY: nuclear Overhauser effect spectroscopy; GC-MS: gas chromatography coupled to mass spectrometry; SPME: solid phase micro extraction; LC-MS: liquid chromatography coupled to mass spectrometry; UPLC: ultra-performance liquid chromatography; ESI: Electrospray ionisation; FID: flame ionisation detection; PFOA: perfluorooctanoic acid; PFCA: perfluorocarboxylic acid; PFDeA: perfluorodecanoic acid; PFUnDA: perfluoroundecanoic acid; EPA: eicoisapentaenoic acid; DPA: docosapentaenoic acid; DHA: docosahexaenoic acid; DGLA: dihomo-gama-linolenic acid; AA: arachidonic acid; OR: odds ratio; CI: confidence interval; NA: not applicable.

For peer review only

Analysis of identified metabolites

The identified compounds refer to eleven HMDB chemical classes. Fatty acids [18,28,39] comprised the most prevalent chemical class, followed by amino acids [18,33] and phosphosphingolipids [17] (Table 3).

A total of 5,974 women were assessed for vitamin D status. Results were presented as total vitamin D, [32,35,37,40] although vitamin D_2, D_3 or 3-epi-25(OH)D_3 [35] metabolites were measured. Results were stratified according to season of maternal sampling or latitude. Either <15ng/mL (<37.5nmol/L) [40] or <20ng/mL (<50nmol/L) [32,35,37] levels characterized vitamin D deficiency, but were statistically different in SGA pregnancies only in the 1st trimester. [32] Horgan et al found a metabolite that could represent a vitamin D derivative, but it was only predictive in combination with 18 other compounds; this model had an area under the curve (AUC) of 0.90 (optimal odds ratio (OR), 44; 95%CI 9-214). [17]

The second most frequent targeted metabolite was homocysteine, [33,34] although levels were only differentiated between normal and SGA pregnancies when measured in 2nd trimester amniotic fluid, with a multiple linear regression model r^2=0.012 and p=0.029. [33] Comparatively, the only common metabolite in 2nd trimester maternal hair was margarate, with conflicting results since it was found to be either increased (AUC 0.72, 95%CI 0.58-0.86) [28] or decreased. [18] The N1,N12-diacetylspermine and the perfluorocarboxylic acids were associated to female SGA babies, not males. The former presented a 5-fold decreased risk of SGA across quintiles. The perfluorodecanoic and perfluoroundecanoic acids presented OR of 3.14 (95%CI 1.07-9.19) and 1.83 (95%CI 1.01-3.32). [36] Tyrosine, an essential amino acid for infants, was part of the predictive model of maternal hair, combining 5 metabolites with an AUC of 0.998 (95%CI 0.992-1.0) [18]. However,

For peer review only

tyrosine did not predict SGA when urine samples were studied. [38] Methylmalonic acid, [34] acetate, formate, or trimethylamine, [38] did not differentiate SGA when compared to uncomplicated pregnancies (p>0.05).

For peer review only

Table 3. Predictive metabolites summarized according to their chemical class, subclass, and biological process

Predictive metabolites	Chemical class	Chemical subclass	Metabolic pathway
Margarate	Fatty acyls	Fatty acids and conjugates	Lipid transport, metabolism, peroxidation
Pentadecanoic acid	Fatty acyls	Fatty acids and conjugates	Lipid transport, metabolism, peroxidation; fatty acid metabolism and biosynthesis
Myristic acid	Fatty acyls	Fatty acids and conjugates	Lipid transport, metabolism, peroxidation; fatty acid metabolism and biosynthesis
Eicosatetraenoic acid	Fatty acyls	Fatty acids and conjugates	Lipid transport, metabolism, peroxidation; lipid metabolism pathway
Docosapentaenoic acid	Fatty acyls	Fatty acids and conjugates	Lipid transport and metabolism, fatty acid metabolism, alpha linolenic acid and linoleic acid metabolisms
Tyrosine [a]	Carboxylic acids and derivatives	Amino acids, peptides, and analogues	Catecholamine biosynthesis; phenylalanine and tyrosine metabolism; thyroid hormone synthesis; transcription and translation
Homocysteine	Carboxylic acids and derivatives	Amino-acids, peptides, and analogues	Glycine and serine metabolism; methionine metabolism
Hexacosanedioic acid	Carboxylic acids and derivatives	Dicarboxylic acid and derivatives	Fatty acid biosynthesis
Sphinganine 1-phosphate	Sphingolipids	Phosphosphingolipids	Sphingolipid signalling pathway, nneuroactive ligand-receptor interaction
Sphingosine 1-phosphate	Sphingolipids	Phosphosphingolipids	Lipid metabolism pathway, sphingolipid metabolism
PFDeA	Alkyl halides	Alkyl fluorides	Not reported[b]
PFUnDA	Alkyl halides	Alkyl fluorides	Not reported[b]
25,OH,Vitamin D	Steroids and steroids derivatives	Vitamin D and derivatives	Lipid metabolism pathway
Diglyceride	Glycerolipids	Diradylglycerols	Adipocytokine signaling pathway
Lactate	Hydroxy acids and derivatives	Alpha hydroxy acids and derivatives	Gluconeogenesis, glycogenosis types IB and IC, pyruvate metabolism, triosephosphate isomerase
N1,N12-diacetylspermine	Carboximidic acids and derivatives	Carboximidic acids	
Lyso-phosphocholine	Glycerophospholipids	Glycerophosphocholines	Not reported[b]
2-methyloctadecanate	Saturated hydrocarbons	Alkanes	Not reported[b]
Levulinate	Keto acids and derivatives	Gamma-keto acids and derivatives	Not reported[b]

[a] Essential amino acid for infants. [b] No human metabolic pathways reported at KEGG. PFDeA: perfluorodecanoic acid; PFUnDA: perfluoroundecanoic acid.

For peer review only

Risk of bias and Applicability Concerns

Figure 2 shows synthesized data for all included studies. See Supplementary Material 2 for individual QUADAS-2 data.

Regarding the risk of bias, all cohort studies conducted a consecutive participant inclusion. [28,33–37,39] Nested case-controls matched cases and controls randomly [33–35,41] or according to maternal and infant characteristics. [17,18,38,42] One study [41] failed to mention matching procedures ('Patient Selection' domain). Researchers were not blinded to SGA status when interpreting metabolomics results, [17,18,41,28,32,35–40] and thresholds of targeted metabolites were not pre-specified [31,33,36,38,39] ('Index Test' domain). Conversely, SGA identification was not influenced by the metabolomics test, although it was unclear when laboratory experiments were performed in some studies. [18,28,31,33,34,41] Birthweight charts were adequate, except for two studies. The first did not report which centile was chosen, [18] and the second used a centile designed for a different population [33] ('Reference Test' domain). Two studies were ranked as 'high risk' because not all participants were included in the analysis [31,37] ('Flow and Timing' domain).

The QUADAS-2 tool also highlights the importance of how the findings of the included studies are suitable to the review question. In the Patient Selection domain, it was ranked as 'high applicability concerns' when infants born between the 4th and the 10th centile, but with normal abdominal circumference growth velocity, were not included in final analysis. [41] It was 'unclear' when the gestational age of maternal assessment was not standardized, [34] or was inferred by hair segment length; [28] or when few metabolites from untargeted studies were chosen for interpretation [41]

For peer review only

('Index Test' domain). Finally, it was 'high' when the birthweight charts applied did not correspond to the study population [18,33] ('Reference Standard' domain).

Meta-analysis

From the 15 included studies, only three were designed for prediction purposes [17,18,42] and provided the AUC. The remaining reports described statistical differences of metabolites between SGA pregnancies and controls. [28,31,40,41,32–39] Accuracy measures were extracted when available (Table 2). However, due to marked heterogeneity (Tables 1 and 2) of gestational age at sampling, type of samples used, type of birthweight chart chosen, thresholds for vitamin D deficiency, metabolomics approach, and identified compounds, a meta-analysis could not be performed.

DISCUSSION

Main findings

In this first systematic review of metabolomics and adverse pregnancy endpoints, we presented techniques and metabolites, which were studied for the prediction of SGA. Any effect on birthweight has important implications for perinatal research, since it is related to short and long-term outcomes, [43–46] and in different generations. [47,48] Intrauterine environment influences fetal growth through epigenetic processes: altered gene expression potentially leads to distinct phenotypes. [49] Metabolomics is the most adequate approach to study this outcome, since it is most directly related to phenotype. [50]

Interpretation of metabolomics findings in pregnancy can be challenging. Firstly, maternal metabolites concentrations are influenced by placental transfer to

For peer review only

and from the fetus. The 'mirror effect', seen for maternal plasma and venous cord blood metabolites at birth, [51] cannot be ruled out when only maternal specimens are studied. Secondly, maternal exposure to distinct compounds may affect metabolite levels. Statistically significant differences between SGA infants and controls may not express the totality of underlying pathological pathways and have no clinical meaning. Finally, it is unclear when the processes leading to SGA are initiated. The disruption in maternal metabolism can theoretically occur at any time. In general the lower the gestational age at which the condition is suspected, the more severe the phenotype will be at birth. [52,53] Thus, the description of clinical data in translational studies must deal with all these confounding factors.

Gestational age at sampling is probably the most important parameter for prediction purposes. With timely prediction, women could be referred to specialized care, have increased surveillance, and this in turn may lead to a reduction in perinatal mortality. There are temporal changes in the maternal metabolome during pregnancy; [28,54–57] therefore, it is reasonable to expect distinctive metabolites at different stages of pregnancy, as reported here. Unfortunately, a wide or unclear definition of gestational age of sampling [34,36,38,40] render a more precise interpretation impossible, and may limit the clinical application of these results.

In contrast, gestational age at birth and birthweight centile seem to be the hallmarks of severity and prognosis of growth restriction. [6,58] Indeed, term and preterm SGA babies show distinct clinical phenotypes, and there are concerns that some babies <10th centile of birthweight are constitutionally small infants. [59–61] If only term deliveries are evaluated, the most severe cases of growth restriction may be potentially missed. Moreover, when term and preterm births are analyzed together, or when lower cutoffs are not specified (e.g. ≤3rd or ≤5th centile), the lack of

For peer review only

predictive metabolites might mean that they are distinct conditions. Thus, we hypothesize that the predictive performance of metabolomics may be improved if data is analyzed by gestational age at delivery, and by different cutoffs of birthweight centiles.

Evidence suggests that tobacco smoke has an impact on birthweight, [62–64] although it is uncertain how and when fetal growth is impaired. It is possibly related to oxidative stress, [65] and both maternal and fetal metabolism may be disturbed at delivery. [66,67] Studies that were included did not investigate cigarette-related chemicals or quantify exposure to tobacco smoke. Therefore, no relationship between SGA and tobacco was found. Hence, we suggest that tobacco interferes with ongoing metabolic pathological processes, or its disturbance is related to additional metabolic pathways other than the one examined by the included studies.

Subgroup and metabolite findings

No reports have explored data on any maternal chronic condition, suspicion of SGA in pregnancy, or number of fetuses. The lack of clear statements about participant selection have hindered data interpretation and precluded these analyses.

The majority of included studies performed a targeted approach, i.e. a hypothesis-testing evaluation, [16,50] driven by epidemiological or experimental data regarding SGA newborns. None of the targeted metabolites [31–40] were in common with those found by ‘hypothesis-generating’ metabolic profiling [17,18,28,41,42] investigations. This reinforces the suggestion that various maternal metabolic pathways may be triggered by the SGA condition, and be detected by different biological samples. However, since blood is a very complex sample and GC-MS only

For peer review only - http://bmjopen.bmj.com/site/about/guidelines.xhtml

evaluates volatile molecules, [50] therefore our findings may be biased by study methodologies.

Untargeted studies, as expected, have characterized several metabolites that may be validated in future investigations. Nine lipids and fatty acid metabolites, [17,18,28,39] two amino acids, [18,33] and a steroid [17,32] have been identified as potential biomarkers of SGA.

All lipid-related metabolites identified are intermediates for energy storage and breakdown. Most metabolites were found in maternal blood [17] or hair of the SGA group. [18,28] Blood levels of saturated and monounsaturated non-esterified fatty acids apparently remain stable throughout pregnancy, while long chain polyunsaturated fatty acid (DHA and EPA, for example) measurements seem to show ethnicity-related changes. [57] Experimental data shows the importance of hypoxia and oxidative stress to placental function and ultimately, to birthweight. [68,69] Findings from included studies may represent a dysregulation of lipid pathways at the placental level, but an association with maternal background is unclear. Therefore, we hypothesize that disorders of lipid metabolism may be the ‘metabolic snapshot’ of defective deep placentation, [70] and might reflect maternal efforts to respond to impaired fetal growth.

Recommendations on the assessment of vitamin D and cutoffs to define vitamin D deficiency in pregnancy are controversial. [71] However, vitamin D supplementation decreases SGA risk. [72] In early pregnancy, vitamin D status has been related to SGA, [73,74] which is in accordance with this review, despite the inconsistent findings. [75] There is evidence that trophoblasts actively produce and secrete vitamin D metabolites, [76] but it is not clear how they mediate fetal growth impairment. Altered hepatic gene expression and liver function in vitamin D deficient

For peer review only

female rats, [77] and single nucleotide polymorphisms [78] in vitamin D receptor gene have been suggested as mechanisms to be explored by a multidimensional omics approach.

Finally, homocysteine is an intermediate metabolite of the folate cycle. It is indirectly involved with DNA methylation and is a marker of folate deficiency. [79] Maternal levels rarely reach hyperhomocysteinemia limits, [80] but folate depletion [81–83] and homocysteine itself[80] are thought to be associated with a higher SGA risk. In this review, homocysteine was only statistically different in SGA pregnancies when measured in amniotic fluid, [33] although within the normal ranges proposed for 17-21 weeks. [84] Since amniocentesis is generally performed in women at higher obstetrical risk, future studies should investigate whether homocysteine in amniotic fluid represents a confounding factor or a new biomarker. [85]

Methodological quality

Most studies were ranked as 'low risk' of bias or applicability to the review question. However, the lack of clear descriptions of laboratory experiments, including sample preparation and storage, and blinding of the researchers to the case/control status, are major pitfalls of the included studies.

Strengths and limitations

To our knowledge, this is the first systematic review of metabolomics and an adverse pregnancy outcome (SGA). We presented possible biomarkers of SGA pathophysiology, metabolites implicated in lipid transport and metabolic pathways, as well as gluconeogenesis.

For peer review only

However, this analysis has some limitations. First, included studies showed heterogeneity, which is fundamental in systematic reviews. Indeed, there was a wide variety of participant characteristics and methods used, and not all authors provided a detailed description of methods employed. Although the Metabolomics Standard Initiative was released in 2007, [86] there is still poor adherence to guidelines. [87,88] Clear reporting [15,87,88] and data sharing in repositories are crucial steps in identifying features of interest, specifically possible biomarkers to be validated in the clinical studies. [15] Secondly, we could not perform a meta-analysis of the extracted data, impacting the translational potential of metabolomics.

Thirdly, we considered that birthweight was a surrogate measure of intrauterine development. SGA and FGR are not interchangeable concepts. However, SGA has been used as a surrogate for FGR in many clinical studies due to difficulties in defining optimal intrauterine growth: (i) FGR diagnosis relies mostly on ultrasound measurements of fetal biometry, [3,89] which in turn is subject to systematic errors; [90] (ii) intrauterine development is adaptive, rather than uniform [91] or only genetically driven; [49] (iii) growth impairment at birth better identifies adverse neonatal outcomes than during pregnancy. [58] It is recognized that changes in obstetric care occur when growth restriction is suspected, and neonatal outcomes are improved. [21,22] Thus, an accurate prediction of SGA during pregnancy will be a turning point in modern obstetrics.

CONCLUSIONS AND IMPLICATIONS FOR PRACTICE

Using the available clinical tools, efforts to predict SGA remain disappointing. Since SGA is a heterogeneous condition, it benefits from metabolomics. This novel area of research allows analysis of numerous types of biological fluids and detects

For peer review only

thousands of metabolites in complex samples. [15,16,25] However, findings of this systematic review must be interpreted with caution. The type of samples used may have influenced LC-MS (2nd trimester maternal blood) and GC-MS (2nd trimester maternal hair) findings in individual studies. Furthermore, the prediction of SGA in the context of maternal disorders, suspected FGR and twin pregnancies is an open field for future metabolomics studies, and environmental exposure investigation as well.

Surprisingly, none of the studies used ≤3rd centile of birthweight as a cutoff or analyzed preterm deliveries and hypertensive syndromes. Considering our findings and the different phenotypic manifestations of SGA, we envision a better performance when (i) cutoffs other than the 10th centile are tested; (ii) data on gestational age at sampling and at birth are standardized; and (iii) other pregnancy-related syndromes are considered, especially hypertension. Thus, future metabolomics results should advance in these critical points.

Finally, all detected biomarkers were related to lipid pathways and energy metabolism. We consider that research efforts to predict SGA should focus on compounds involved in these pathways, up to the 2nd trimester of pregnancy.

For peer review only

REFERENCES

1 American College of Obstetricians and Gynecologists (ACOG). Practice Bulletin 134. Fetal Growth Restriction. *Obstet Gynecol* 2013;**121**:1122–33.

2 Figueras F, Gratacós E. Update on the diagnosis and classification of fetal growth restriction and proposal of a stage-based management protocol. *Fetal Diagn Ther* 2014;**36**:86–98. doi:10.1159/000357592

3 Gordijn SJ, Beune IM, Thilaganathan B, *et al.* Consensus definition of fetal growth restriction: a Delphi procedure. *Ultrasound Obstet Gynecol* 2016;**48**:333–9. doi:10.1002/uog.15884

4 Bukowski R, Hansen NI, Willinger M, *et al.* Fetal Growth and Risk of Stillbirth: A Population-Based Case-Control Study. *PLoS Med* 2014;**11**. doi:10.1371/journal.pmed.1001633

5 Lee ACC, Katz J, Blencowe H, *et al.* National and regional estimates of term and preterm babies born small for gestational age in 138 low-income and middle-income countries in 2010. *Lancet Glob Heal* 2013;**1**:e26-36. doi:10.1016/S2214-109X(13)70006-8

6 Mendez-Figueroa H, Truong VTT, Pedroza C, *et al.* Small-for-gestational-age infants among uncomplicated pregnancies at term: a secondary analysis of 9 Maternal-Fetal Medicine Units Network studies. *Am J Obstet Gynecol* 2016;**215**:628.e1-628.e7. doi:10.1016/j.ajog.2016.06.043

7 Sharma D, Farahbakhsh N, Shastri S, *et al.* Intrauterine growth restriction–part 2. *J Matern Neonatal Med* 2016;**29**:4037–48. doi:10.3109/14767058.2016.1154525

8 Barker DP, Osmond C, Simmonds SJ, *et al.* The relation of small head circumference and thinness at birth to death from cardiovascular disease in adult life. *Br Med J* 1993;**306**:422–6. doi:10.1136/bmj.306.6875.422

9 Balasuriya CND, Stunes AK, Mosti MP, *et al.* Metabolic Outcomes in Adults Born Preterm with Very Low Birth Weight or Small for Gestational Age at Term: A Cohort Study. *J Clin Endocrinol Metab* 2018;**103**:4437–46. doi:10.1210/jc.2018-00464

10 Goto E. Maternal anthropometry to predict small for gestational age: A meta-analysis. *Eur J Obstet Gynecol Reprod Biol* 2016;**203**:193–8. doi:10.1016/j.ejogrb.2016.05.042

11 Pay ASD, Frøen JF, Staff AC, *et al.* Prediction of small-for-gestational-age status by symphysis–fundus height: a registry-based population cohort study. *BJOG* 2016;**123**:1167–73. doi:10.1111/1471-0528.13727

12 Parry S, Sciscione A, Haas DM, *et al.* Role of early second-trimester uterine artery Doppler screening to predict small-for-gestational-age babies in nulliparous women. *Am J Obstet Gynecol* 2017;**217**:594.e1-594.e10. doi:10.1016/j.ajog.2017.06.013

13 Roma E, Arnau A, Berdala R, *et al.* Ultrasound screening for fetal growth restriction at 36 vs 32 weeks' gestation: A randomized trial (ROUTE). *Ultrasound Obstet Gynecol* 2015;**46**:391–7. doi:10.1002/uog.14915

14 Conde-Agudelo A, Papageorghiou a T, Kennedy SH, *et al.* Novel biomarkers for predicting intrauterine growth restriction: a systematic review and meta-analysis. *BJOG* 2013;**120**:681–94. doi:10.1111/1471-0528.12172

15 Xia J, Broadhurst DI, Wilson M, *et al.* Translational biomarker discovery in

For peer review only

clinical metabolomics: An introductory tutorial. *Metabolomics* 2013;**9**:280–99. doi:10.1007/s11306-012-0482-9

16 Patti GJ, Yanes O, Siuzdak G. Metabolomics: The apogee of the omics trilogy. *Nat Rev Mol cell Biol* 2012;**13**:263–9. doi:10.1038/nrm3314

17 Horgan RP, Broadhurst DI, Walsh SK, *et al.* Metabolic profiling uncovers a phenotypic signature of small for gestational age in early pregnancy. *J Proteome Res* 2011;**10**:3660–73.

18 Sulek K, Han TL, Villas-Boas SG ranat., *et al.* Hair metabolomics: identification of fetal compromise provides proof of concept for biomarker discovery. *Theranostics* 2014;**4**:953–9. doi:10.7150/thno.9265

19 Favretto D, Cosmi E, Ragazzi E, *et al.* Cord blood metabolomic profiling in intrauterine growth restriction. *Anal Bioanal Chem* 2012;**402**:1109–21. doi:10.1007/s00216-011-5540-z

20 Sanz-Cortés M, Carbajo RJ, Crispi F, *et al.* Metabolomic profile of umbilical cord blood plasma from early and late intrauterine growth restricted (IUGR) neonates with and without signs of brain vasodilation. *PLoS One* 2013;**8**:e80121. doi:10.1371/journal.pone.0080121

21 Monier I, Blondel B, Ego A, *et al.* Does the Presence of Risk Factors for Fetal Growth Restriction Increase the Probability of Antenatal Detection? A French National Study. *Paediatr Perinat Epidemiol* 2016;**30**:46–55. doi:10.1111/ppe.12251

22 Verlijsdonk JW, Winkens B, Boers K, *et al.* Suspected versus non-suspected small-for-gestational age fetuses at term: Perinatal outcomes. *J Matern Neonatal Med* 2012;**25**:938–43. doi:10.3109/14767058.2011.600793

23 Leite DFB, Morillon A-C, Melo Júnior EF, *et al.* Metabolomics for predicting fetal growth restriction: protocol for a systematic review and meta-analysis. *BMJ Open* 2018;**8**:e022743. doi:10.1136/bmjopen-2018-022743

24 Moher D, Liberati A, Tetzlaff J, *et al.* Preferred Reporting Items for Systematic Reviews and Meta-Analyses: the PRISMA statement. *Ann Intern Med* 2009;**151**:264–9. doi:10.1371/journal.pmed1000097

25 Wishart DS, Feunang YD, Marcu A, *et al.* HMDB 4.0: The human metabolome database for 2018. *Nucleic Acids Res* 2018;**46**:D608–17. doi:10.1093/nar/gkx1089

26 Kyoto Encyclopedia of Genes and Genomes. https://www.genome.jp/kegg/ (accessed 20 Dec 2018).

27 Whiting PF, Rutjes AWS, Westwood ME, *et al.* Research and Reporting Methods Accuracy Studies. *Ann Intern Med* 2011;**155**:529–36. doi:10.7326/0003-4819-155-8-201110180-00009

28 Delplancke TDJ, De Seymour J V., Tong C, *et al.* Analysis of sequential hair segments reflects changes in the metabolome across the trimesters of pregnancy. *Sci Rep* 2018;**8**:1–12. doi:10.1038/s41598-017-18317-7

29 Rutter CM, Gatsonis CA. A hierarchical regression approach to meta-analysis of diagnostic test accuracy evaluations. *Stat Med* 2001;**20**:2865–84. doi:10.1002/sim.942

30 Deeks JJ, Macaskill P, Irwig L. The performance of tests of publication bias and other sample size effects in systematic reviews of diagnostic test accuracy was assessed. *J Clin Epidemiol* 2005;**58**:882–93. doi:10.1016/j.jclinepi.2005.01.016

31 Costet N, Garlantézec R, Monfort C, *et al.* Environmental and urinary markers of prenatal exposure to drinking water disinfection by-products, fetal growth,

For peer review only

and duration of gestation in the PELAGIE birth cohort (Brittany, France, 2002-2006). *Am J Epidemiol* 2012;**175**:263–75. doi:10.1093/aje/kwr419

32 Ertl R, Yu CKH, Samaha R, *et al.* Maternal serum vitamin D at 11-13 weeks in pregnancies delivering small for gestational age neonates. *Fetal Diagn Ther* 2012;**31**:103–8. doi:10.1159/000333810

33 Grandone E, Colaizzo D, Vecchione G, *et al.* Homocysteine levels in amniotic fuild. *Thromb Haemost* 2006;**95**:625–8. doi:10.1160/TH05

34 Choi R, Choi S, Lim Y, *et al.* A prospective study on serum methylmalonic acid and homocysteine in pregnant women. *Nutrients* 2016;**8**:1–15. doi:10.3390/nu8120797

35 Kiely ME, Zhang JY, Kinsella M, *et al.* Vitamin D status is associated with uteroplacental dysfunction indicated by pre-eclampsia and small-for-gestational-age birth in a large prospective pregnancy cohort in Ireland with low Vitamin D status. *Am J Clin Nutr* 2016;**104**:354–61. doi:10.3945/ajcn.116.130419

36 Wang Y, Adgent M, Su PH, *et al.* Prenatal exposure to perfluorocarboxylic acids (PFCAs) and fetal and postnatal growth in the Taiwan maternal and infant cohort study. *Environ Health Perspect* 2016;**124**:1794–800. doi:10.1289/ehp.1509998

37 Ong YL, Quah PL, Tint MT, *et al.* The association of maternal Vitamin D status with infant birth outcomes, postnatal growth and adiposity in the first 2 years of life in a multi-ethnic Asian population: The Growing Up in Singapore Towards healthy Outcomes (GUSTO) cohort study. *Br J Nutr* 2016;**116**:621–31. doi:10.1017/S0007114516000623

38 Luthra G, Vuckovic I, Bangdiwala A, *et al.* First and second trimester urinary metabolic profiles and fetal growth restriction: An exploratory nested case-control study within the infant development and environment study. *BMC Pregnancy Childbirth* 2018;**18**:1–8. doi:10.1186/s12884-018-1674-8

39 van Eijsden M, Hornstra G, van der Wal MF, *et al.* Maternal n-3, n-6, and trans fatty acid profile early in pregnancy and term birth weight: a prospective cohort study. *Am J Clin Nutr* 2008;**87**:887–95.

40 Gernand AD, Simhan HN, Klebanoff MA, *et al.* Maternal serum 25-hydroxyvitamin D and measures of newborn and placental weight in a U.S. multicenter cohort study. *J Clin Endocrinol Metab* 2013;**98**:398–404. doi:10.1210/jc.2012-3275

41 Gong S, Sovio U, Aye ILMH, *et al.* Placental polyamine metabolism differs by fetal sex, fetal growth restriction, and preeclampsia. *JCI Insight* 2018;**3**:1–15. doi:10.1172/jci.insight.120723

42 Morillon A-C, Yakkundi S, Thomas G, *et al.* Untargeted UPLC-MS analysis of potential pesticide and biomarkers of fetal growth restriction. *Conf Proceedings 14th Annu Conf Metabolomics Soc* 2018;:200.

43 Wang N, Wang X, Li Q, *et al.* The famine exposure in early life and metabolic syndrome in adulthood. *Clin Nutr* 2017;**36**:253–9. doi:10.1016/j.clnu.2015.11.010

44 Hales CN, Barker DJP. The thrifty phenotype hypothesis. *Br Med Bull* 2001;**60**:5–20. doi:10.1093/bmb/60.1.5

45 Melo AS, Vieira CS, Barbieri MA, *et al.* High prevalence of polycystic ovary syndrome in women born small for gestational age. *Hum Reprod* 2010;**25**:2124–31. doi:10.1093/humrep/deq162

46 Ravelli a CJ, Vandermeulen JHP, Michels RPJ, *et al.* Glucose-tolerance in

For peer review only - http://bmjopen.bmj.com/site/about/guidelines.xhtml

adults after prenatal exposure to famine. *Lancet* 1998;**351**:173–7. doi:10.1016/S0140-6736(97)07244-9

47 Chamorro-Garcia R, Diaz-Castillo C, Shoucri BM, *et al.* Ancestral perinatal obesogen exposure results in a transgenerational thrifty phenotype in mice. *Nat Commun* 2017;**8**. doi:10.1038/s41467-017-01944-z

48 Seferovic MD, Goodspeed DM, Chu DM, *et al.* Heritable IUGR and adult metabolic syndrome are reversible and associated with alterations in the metabolome following dietary supplementation of 1-carbon intermediates. *FASEB J* 2015;**29**:2640–52. doi:10.1096/fj.14-266387

49 Padmanabhan V, Cardoso RC, Puttabyatappa M. Developmental programming, a pathway to disease. *Endocrinology* 2016;**157**:1328–40. doi:10.1210/en.2016-1003

50 Dunn WB, Broadhurst DI, Atherton HJ, *et al.* Systems level studies of mammalian metabolomes: The roles of mass spectrometry and nuclear magnetic resonance spectroscopy. *Chem Soc Rev* 2011;**40**:387–426. doi:10.1039/b906712b

51 Visentin S, Crotti S, Donazzolo E, *et al.* Medium chain fatty acids in intrauterine growth restricted and small for gestational age pregnancies. *Metabolomics* 2017;**13**:1–9. doi:10.1007/s11306-017-1197-8

52 Korzeniewski SJ, Allred EN, Joseph RM, *et al.* Neurodevelopment at age 10 years of children born <28 weeks with fetal growth restriction. *Pediatrics* 2017;**140 (5) (n**. doi:http://dx.doi.org/10.1542/peds.2017-0697

53 Lees C, Marlow N, Arabin B, *et al.* Perinatal morbidity and mortality in early-onset fetal growth restriction: Cohort outcomes of the trial of randomized umbilical and fetal flow in Europe (TRUFFLE). *Ultrasound Obstet Gynecol* 2013;**42**:400–8. doi:10.1002/uog.13190

54 Luan H, Meng N, Liu P, *et al.* Non-targeted metabolomics and lipidomics LC-MS data from maternal plasma of 180 healthy pregnant women. *Gigascience* 2015;**4**:16–9. doi:10.1186/s13742-015-0054-9

55 Di Giulio AM, Carelli S, Castoldi RE, *et al.* Plasma amino acid concentrations throughout normal pregnancy and early stages of intrauterine growth restricted pregnancy. *J Matern Neonatal Med* 2004;**15**:356–62. doi:10.1080/14767050410001725578

56 Orczyk-Pawilowicz M, Jawien E, Deja S, *et al.* Metabolomics of human amniotic fluid and maternal plasma during normal pregnancy. *PLoS One* 2016;**11**:1–13. doi:10.1371/journal.pone.0152740

57 Lindsay KL, Hellmuth C, Uhl O, *et al.* Longitudinal metabolomic profiling of amino acids and lipids across healthy pregnancy. *PLoS One* 2015;**10**:1–19. doi:10.1371/journal.pone.0145794

58 Boghossian NS, Geraci M, Edwards EM, *et al.* Neonatal and fetal growth charts to identify preterm infants <30 weeks gestation at risk of adverse outcomes. *Am J Obstet Gynecol* 2018;**219**:195.e1-195.e14. doi:10.1016/j.ajog.2018.05.002

59 Katz J, Wu LA, Mullany LC, *et al.* Prevalence of small-for-gestational-age and its mortality risk varies by choice of birth-weight-for-gestation reference population. *PLoS One* 2014;**9**:1–9. doi:10.1371/journal.pone.0092074

60 Ray JG, Jiang D, Sgro M, *et al.* Thresholds for Small for Gestational Age Among Newborns of East Asian and South Asian Ancestry. *J Obstet Gynaecol Canada* 2009;**31**:322–30. doi:10.1016/S1701-2163(16)34149-4

61 Cheng YKY, Leung TY, Lao TTH, *et al.* Impact of replacing Chinese ethnicity-

For peer review only

specific fetal biometry charts with the INTERGROWTH-21ststandard. *BJOG An Int J Obstet Gynaecol* 2016;**123**:48–55. doi:10.1111/1471-0528.14008

62 Abraham M, Alramadhan S, Iniguez C, *et al.* A systematic review of maternal smoking during pregnancy and fetal measurements with meta-analysis. *PLoS One* 2017;**12**:1–13. doi:10.1371/journal.pone.0170946

63 Vesterinen HM, Morello-Frosch R, Sen S, *et al.* Cumulative effects of prenatal-exposure to exogenous chemicals and psychosocial stress on fetal growth: Systematic-review of the human and animal evidence. *PLoS One* 2017;**12**. doi:10.1371/journal.pone.0176331

64 Dessì A, Corona L, Pintus R, *et al.* Exposure to tobacco smoke and low birth weight: from epidemiology to metabolomics. *Expert Rev Proteomics* 2018;**15**:647–56. doi:10.1080/14789450.2018.1505508

65 Stone WL, Bailey B, Khaisha N. The pathophysiology of smoking during pregnancy: a systems biology approach. *Front Biosci* 2014;**E6**:318–28.

66 Fischera T, Lilic LN, Lic S, *et al.* Low-level maternal exposure to nicotine associates with significant metabolic perturbations in second-trimester amniotic fluid. *Env Int* 2017;**107**:227–34. doi:10.1001/jamasurg.2014.1086.Feasibility

67 Rolle-Kampczyk UE, Krumsiek J, Otto W, *et al.* Metabolomics reveals effects of maternal smoking on endogenous metabolites from lipid metabolism in cord blood of newborns. *Metabolomics* 2016;**12**. doi:10.1007/s11306-016-0983-z

68 Thomas MM, Haghiac M, Grozav C, *et al.* Oxidative Stress Impairs Fatty Acid Oxidation and Mitochondrial Function in the Term Placenta. *Reprod Sci* 2018;:193371911880205. doi:10.1177/1933719118802054

69 Määttä J, Sissala N, Dimova EY, *et al.* Hypoxia causes reductions in birth weight by altering maternal glucose and lipid metabolism. *Sci Rep* 2018;**8**:13583. doi:10.1038/s41598-018-31908-2

70 Brosens I, Pijnenborg R, Vercruysse L, *et al.* The ‘great obstetrical syndromes’ are associated with disorders of deep placentation. *Am J Obs Gynecol* 2011;**204**:193–201. doi:10.1016/j.ajog.2010.08.009.THE

71 Holick MF, Binkley NC, Bischoff-Ferrari HA, *et al.* Evaluation, treatment, and prevention of vitamin D deficiency: An endocrine society clinical practice guideline. *J Clin Endocrinol Metab* 2011;**96**:1911–30. doi:10.1210/jc.2011-0385

72 Bi WG, Nuyt AM, Weiler H, *et al.* Association between Vitamin D supplementation during pregnancy and offspring growth, morbidity, and mortality: A systematic review and meta-analysis. *JAMA Pediatr* 2018;**172**:635–45. doi:10.1001/jamapediatrics.2018.0302

73 Bodnar LM, Catov JM, Zmuda JM, *et al.* Maternal Serum 25-Hydroxyvitamin D Concentrations Are Associated with Small-for-Gestational Age Births in White Women. *J Nutr* 2010;**140**:999–1006. doi:10.3945/jn.109.119636.pregnancy

74 Leffelaar ER, Vrijkotte TGM, Van Eijsden M. Maternal early pregnancy vitamin D status in relation to fetal and neonatal growth: Results of the multi-ethnic Amsterdam Born Children and their Development cohort. *Br J Nutr* 2010;**104**:108–17. doi:10.1017/S000711451000022X

75 Martínez-Domínguez SJ, Tajada M, Chedraui P, *et al.* Systematic review and meta-analysis of Spanish studies regarding the association between maternal 25-hydroxyvitamin D levels and perinatal outcomes. *Gynecol Endocrinol* 2018;:1–8. doi:10.1080/09513590.2018.1472761

76 Park H, Wood MR, Malysheva O V., *et al.* Placental Vitamin D metabolism and

its associations with circulating Vitamin D metabolites in pregnant women. *Am J Clin Nutr* 2017;**106**:1439–48. doi:10.3945/ajcn.117.153429

77 Sharma SS, Jangale NM, Harsulkar AM, *et al. Chronic maternal calcium and 25-hydroxyvitamin D deficiency in Wistar rats programs abnormal hepatic gene expression leading to hepatic steatosis in female offspring*. 2017. doi:10.1016/j.jnutbio.2017.01.008

78 Barchitta M, Maugeri A, La Rosa MC, *et al.* Single nucleotide polymorphisms in vitamin D receptor gene affect birth weight and the risk of preterm birth: Results from the “mamma & bambino” cohort and a meta-analysis. *Nutrients* 2018;**10**:18. doi:10.3390/nu10091172

79 Kim J, Kim H, Roh H, *et al.* Causes of hyperhomocysteinemia and its pathological significance. *Arch Pharm Res* 2018;**41**:372–83. doi:10.1007/s12272-018-1016-4

80 Yajnik CS, Chandak GR, Joglekar C, *et al.* Maternal homocysteine in pregnancy and offspring birthweight: Epidemiological associations and Mendelian randomization analysis. *Int J Epidemiol* 2014;**43**:1487–97. doi:10.1093/ije/dyu132

81 Smits LJM, Essed GGM. Short interpregnancy intervals and unfavourable pregnancy outcome: Role of folate depletion. *Lancet* 2001;**358**:2074–7. doi:10.1016/S0140-6736(01)07105-7

82 van Eijsden M, van der Wal MF, Bonsel GJ. Association between short interpregnancy intervals and term birth. *Am J Clin Nutr* 2008;**88**:147–53. doi:10.1016/0013-4694(81)90034-1

83 Hogeveen M, Blom HJ, Den Heijer M. Maternal homocysteine and small-for-gestational-age offspring: Systematic review and meta-analysis. *Am J Clin Nutr* 2012;**95**:130–6. doi:10.3945/ajcn.111.016212

84 Imbard A, Blom HJ, Schlemmer D, *et al.* Methylation metabolites in amniotic fluid depend on gestational age. *Prenat Diagn* 2013;**33**:848–55. doi:10.1002/pd.4142

85 Heazell A, Newman L, Lean S, *et al.* Pregnancy outcome in mothers over the age of 35. *Curr Opin Obs Gynecol* 2018;**30**:337–43.

86 Goodacre R, Broadhurst D, Smilde AK, *et al.* Proposed minimum reporting standards for data analysis in metabolomics. *Metabolomics* 2007;**3**:231–41. doi:10.1007/s11306-007-0081-3

87 Considine EC, Thomas G, Boulesteix AL, *et al.* Critical review of reporting of the data analysis step in metabolomics. *Metabolomics* 2018;**14**. doi:10.1007/s11306-017-1299-3

88 Spicer RA, Salek R, Steinbeck C. Compliance with minimum information guidelines in public metabolomics repositories. *Sci Data* 2017;**4**:1–8. doi:10.1038/sdata.2017.137

89 McCowan LM, Figueras F, Anderson NH. Evidence-based national guidelines for the management of suspected fetal growth restriction: comparison, consensus, and controversy. *Am J Obstet Gynecol* 2018;**218**:S855–68. doi:10.1016/j.ajog.2017.12.004

90 Lappen JR, Myers SA. The systematic error in the estimation of fetal weight and the underestimation of fetal growth restriction. *Am J Obstet Gynecol* 2017;**216**:477–83. doi:10.1016/j.ajog.2017.02.013

91 Papageorghiou AT, Ohuma EO, Altman DG, *et al.* International standards for fetal growth based on serial ultrasound measurements: The Fetal Growth Longitudinal Study of the INTERGROWTH-21st Project. *Lancet*

For peer review only

2014;**384**:869–79. doi:10.1016/S0140-6736(14)61490-2

For peer review only

AUTHORS CONTRIBUTIONS

DFBL and ACM have equally contributed to this report, and both are guarantors of this review. They elaborated the protocol, searched the literature, selected studies, extracted data, assessed risk of bias, and drafted the initial manuscript. RTS and EFMJ have participated in judging inclusion of studies, interpreting data, and revising the manuscript. FM have supported data extraction and have critically examined the clinical interpretation of results. ASK has discussed the quantitative data synthesis, and supervised the report writing. PNB, LCK, and JGC have supervised and approved all steps. All authors have read and agree with this submission.

ACKNOWLEDGEMENTS

We are grateful to (i) Shauna Barret, from the Brookfield Library, University College Cork, Ireland, for her support with the literature search; (ii) Ting-Li Han, from the Department of Obstetrics, The First Affiliated Hospital of Chongqing Medical University, China, for providing additional data for this systematic review; (iii) and Luis Felipe D'Orsi, from University of Campinas, for his support with methods' issues.

FUNDING

DFBL (process number 88881.134512/2016-01) and RTS (88881.134095/2016-01) have awarded scholarships from Brazilian Federal Agency for Support and Evaluation of Graduate Education (CAPES). ACM was granted a scholarship from Science Foundation Ireland, for her doctoral thesis. PRETERM-SAMBA has granted sponsor from Brazilian National Research Council (CNPq) (Award 401636/2013-5) and from the Bill and Melinda Gates Foundation (grant OPP1107597), corresponding

For peer review only

to the research call “Grand Challenges Brazil: Reducing the burden of preterm birth”, number 05/2013. This research received no specific grant from commercial or not-for-profit sectors. Our sponsors have not intervened in authors’ decision to write the systematic review protocol or to submit this paper.

COMPETING INTERESTS

None to declare.

PROVENANCE AND PEER REVIEW

Not commissioned; externally peer reviewed.

Figure captions

Figure 1. PRISMA flowchart of study identification, screening and selection. From: Moher D, Liberati A, Tetzlaff J, Altman DG, The PRISMA Group (2009). Preferred Reporting Items for Systematic Reviews and Meta-Analyses: The PRISMA Statement. PLoS Med 6(7): e1000097. doi:10.1371/journal.pmed1000097. For more information, visit www.prisma-statement.org.

Figure 2. Assessment of risk of bias (A) and applicability concerns (B) of individual studies.

Supplementary material description

Supplementary material 1 – List of excluded studies and reasons.

Supplementary material 2 - Individual QUADAS-2 data for all 15 included studies.

For peer review only

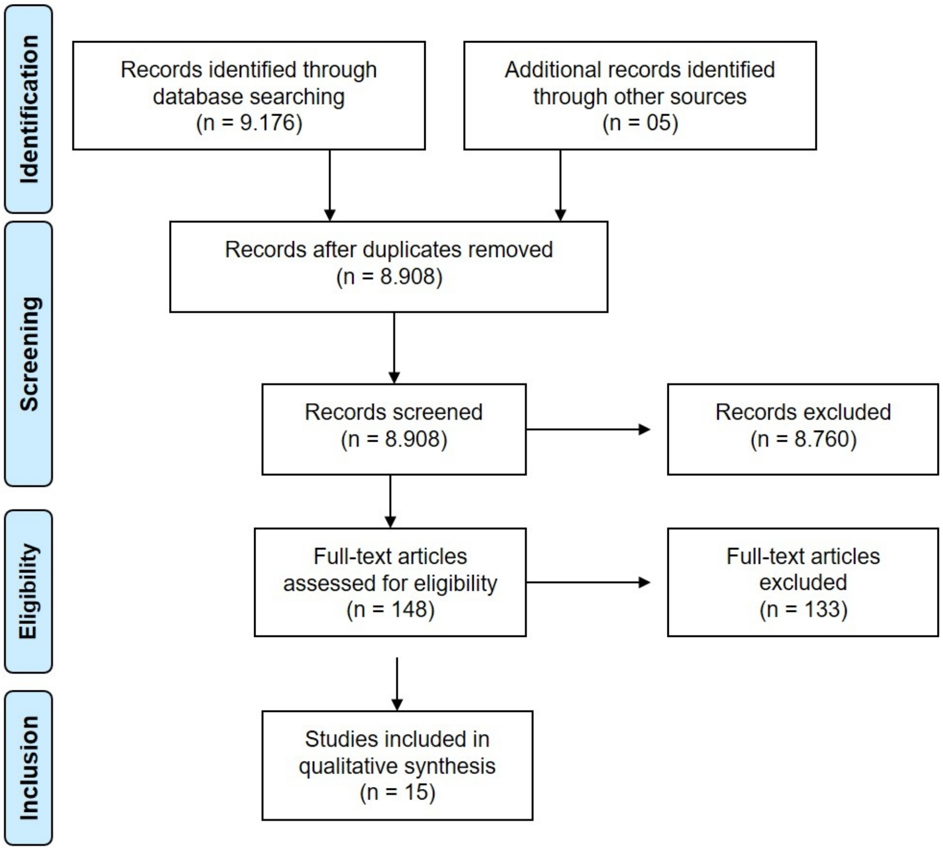

Figure 1

176x155mm (150 x 150 DPI)

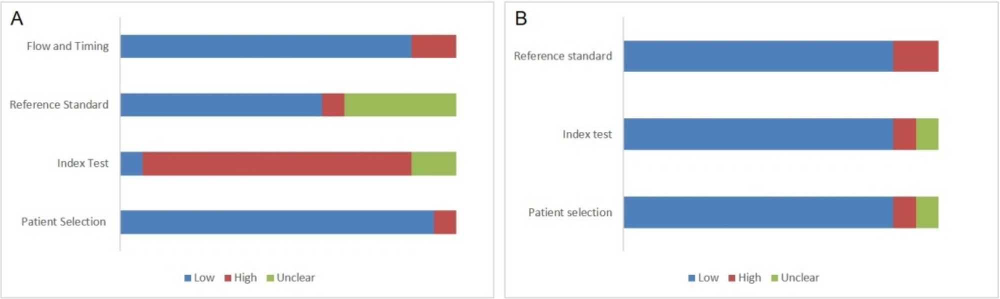

Figure 2

257x76mm (150 x 150 DPI)

Examining the predictive accuracy of metabolomics for small for gestational age babies: a systematic review

Debora F. B. Leite & Aude-Claire Morillon, Elias F. Melo Júnior, Renato T. Souza, Fergus P. McCarthy, Ali S. Khashan, Philip N. Baker, Louise C. Kenny, José Guilherme Cecatti.

Supplementary material 1 – List of excluded studies and reasons.

For peer review only

Authors/ year	Country of enrollment	Additional comments
Exclusions according to study design or statistical analysis		
Barnes CM et al, 2010	United States	Maternal samples collected at delivery.
Bobinski R. 2013	Poland	Cross-sectional study.
Bobinski R. 2014	Poland	Cross-sectional study.
Cao WC et al, 2016	China	Cross-sectional study. The metabolomics technique was not applied.
Chen TT et al, 2017	China	Cross-sectional study.
Cinelli et al, 2018	Italy	
D'Anna R et al, 2004	Italy	Cross-sectional study. The metabolomics technique was not applied.
Guo H et al, 2014	China	Cross-sectional study.

Guo J et al, 2016	China	Cross-sectional study.
Maekawa R et al, 2017	Japan	Cross-sectional study.
Mao D et al, 2010	China	Cross-sectional study.
Miranda J et al 2018	Spain	Cross-sectional study.
Powell et al, 2018	Australia	SGA babies not suspected before birth were considered healthy infants.
Spanou L. et al, 2017	Greece	Cross-sectional study.
Stein TP et al, 2008	United States	Newborns with birth defects were included in the analysis.
Tang R et al, 2013	China	Cross-sectional study.
Visentin S et al, 2017	Italy	Maternal samples collected after clinical recognition of FGR/SGA.
Zhu Y et al, 2018	China	Cross-sectional study.
Zota AR et al, 2009	United States	Cross-sectional study. The metabolomics technique was not applied.
Studies that have not applied metabolomics technique		
Baker PN, 2009	United Kingdom	
Berkowitz GS et al, 2004	United States	
Bodnar LM et al, 2012	United States	

For peer review only

Braun JM et al, 2011	United States	There is no data about FGR.
Cetin I et al, 2002	Italy	
Chong MFF et al, 2015	Singapore	There is no data about birth weight.
Colapinto CK et al, 2015	Canada	The metabolomics technique was not applied for pregnant women's specimens.
Cupul-Uicab LA et al, 2013	United States	
Fruscalzo A et al, 2015	Italy	There is no data about birth weight.
Jusko TA et al, 2006	United States	
Koepke R et al, 2004	Mexico	
Lõpez-Alarcõn M et al, 2015	Mexico	There is no data about birth weight.
Maruta E et al, 2017	Japan	
Miranda ML et al, 2015	United States	
Morley R et al, 2006	Australia	
Muthayya S et al, 2006	India	
Paşaoğlu H et al, 2003	Turkey	
Rahman A et al, 2009	Bangladesh	
Rajasingam D et al, 2009	United Kingdom	

For peer review only

Savitz DA et al, 2002	United States	The metabolomics technique was not applied for pregnant women's specimens.
Savvidou MD et al, 2003	United Kingdom	
Schneuer FJ et al, 2014	Australia	
Snijder CA et al, 2013	Netherlands	
Sweeney AM & Symanski E, 2007	United States	
Takimoto H et al, 2007	Japan	
Terrell ML et al, 2015	United States	
Wei Y et al, 2017	Bangladesh	
Weisskopf MG et al, 2005	United States	
Whyatt RM et al, 2009	United States	
Xue F et al, 2007	United States	
Studies that have not presented specific data about FGR/SGA		
Bach CC et al, 2016	Denmark	
Bachkangi P et al.	United Kingdom	
Bahado-Singh RO et al, 2012	United Kingdom	

For peer review only

Bahado-Singh RO et al, 2015	United Kingdom
Bahado-Singh RO et al, 2017	United Kingdom
Bentley-Lewis R, 2015	United States
Braun JM et al, 2009	United States
Buckley JP et al, 2016	United States
Cantonwine D et al, 2010	Mexico
Cantonwine D et al, 2015	United States
Casas M et al, 2016	Spain
Castorina R et al, 2017 (a)	United States
Chou WC et al, 2014.	Taiwan
Cunha Figueiredo AC et al, 2017	Brazil
Dalsager L et al, 2018	Denmark
De Renzy-Martin KT. et al, 2014	Poland
Debost-Legrand A et al, 2016	France
Desert et al, 2015	France

For peer review only

Diaz SO et al, 2011	Portugal
Diaz SO et al, 2013	Portugal
Dobierzewska A et al, 2017	Chile
Dudzik D et al, 2015	Spain.
Engström KS et al, 2010	Bangladesh
Ettinger AS et al, 2017	Canada
Feng L et al, 2016	China
Ferguson KK et al, 2014	United States
Ferguson KK et al, 2015	United States
Ferguson KK et al, 2017	United States
Finkelstein JL et al, 2015	United States
Fischer ST et al, 2017	United States
Gao H et al, 2017	China
Gardner RM et al, 2011	Bangladesh
Ghartey J et al, 2017	United States
Graça G et al, 2010	Portugal

For peer review only

Graça G et al, 2012	Portugal	
Graça G et al, 2012 (b)	Portugal	
Hogeveen M et al, 2010	Netherlands	
Huang J et al, 2017	China	
Kalhan SC et al, 2003	United States	
Khalil AA et al, 2013	United Kingdom	
Kuc S et al, 2014	Netherlands	
Lenters V et al, 2013	Greenland, Poland, Ukraine	
Lenters V et al, 2016	Greenland, Poland, Ukraine	
Liu K et al, 2017	China	
Lopez-Espinosa MJ et al, 2015	Spain	
Marchlewicz EH et al, 2016	United States	
Minatoya M et al, 2017	Japan	
Minatoya M et al, 2017 (b)	Japan	
Minatoya M et al, 2018	Japan	
Murphy MM et al, 2007	Spain	There is no data about any pregnancy outcomes.

For peer review only

Odibo AO et al, 2011	United States	
Pinney SE et al, 2017	United States	
Polanska K et al, 2014	Poland	
Polanska K et al, 2014 (b)	Poland	
Porter A et al, 2018	United States	
Rejc B et al, 2016	Slovenia	
Rijvers CAH et al, 2013	Netherlands	
Robledo C et al, 2013	United States	
Sachse D et al, 2012	Norway	
Scholtens DM et al, 2016	United Kingdom	
Shisler S et al, 2017	United States	Not all analysis were performed with metabolomics approach.
Tamblyn JA et al, 2018	Ireland	Duplicate data. Check Kiely ME et al, 2016.
Thomas MM et al, 2015	New Zealand	
Van Lee L et al, 2015	Singapore	
Virgiliou C et al, 2017	Greece	
Walsh J et al, 2012	Ireland	

For peer review only

Wang PW et al, 2015	Taiwan	
Watkins DJ et al, 2016	United States	
Wolff MS et al, 2008	United States	
Woods MM et al, 2017	United States	
Yang P et al, 2018	China	
Duplicate data		
Horgan R et al, 2009	Australia	Check Horgan R et al, 2011.
Horgan R et al, 2011	Australia	Check Horgan R et al, 2011.
Khashan AS et al, 2013	Ireland	Check Kiely ME et al, 2016.
Sulek et al, 2014	Singapore	Check Sulek et al, 2014.

For peer review only

Examining the predictive accuracy of metabolomics for small for gestational age babies: a systematic review

Debora F. B. Leite & Aude-Claire Morillon, Elias F. Melo Júnior, Renato T. Souza, Fergus P. McCarthy, Ali S. Khashan, Philip N. Baker, Louise C. Kenny, José Guilherme Cecatti.

Supplementary material 2 - Individual QUADAS-2 data for all 15 included studies.

**Studies**	**Risk of bias**							
	**Patient selection**		**Index test**		**Reference standard**		**Flow and timing**	
	Was a consecutive or random sample of patients enrolled?	Did the study avoid inappropriate exclusions?	Were the index test results interpreted without knowledge of the results of the reference standard?	If a threshold was used, was it pre-specified?	Is the reference standard likely to correctly classify the target condition?	Were the reference standard results interpreted without knowledge of the results of the index test?	Did all patients receive the same reference standard?	Were all patients included in the analysis?
Grandone E et al, 2006	Yes	Yes	Unclear	No	No	Unclear	Yes	Yes
van Eijsden M et al, 2008	Yes	Yes	No	No	Yes	Yes	Yes	Yes
Horgan R et al, 2011	Yes	Yes	No	Yes	Yes	Yes	Yes	Yes
Costet N et al, 2012	Yes	Yes	Yes	No	Yes	Unclear	Yes	No
Ertl R et al, 2012	Yes	Yes	No	Yes	Yes	Yes	Yes	Yes
Gernand AD et al, 2013	Yes	Yes	No	Yes	Yes	Yes	Yes	Yes
Sulek K et al, 2014	Yes	Yes	No	Yes	Unclear	Unclear	Yes	Yes
Choi R et al, 2016	Yes	Yes	Unclear	Yes	Yes	Unclear	Yes	Yes
Kiely ME et al, 2016	Yes	Yes	No	Yes	Yes	Yes	Yes	Yes
Ong YL et al, 2016	Yes	Yes	No	Yes	Yes	Yes	Yes	No
Wang Y et al, 2016	Yes	Yes	No	No	Yes	Yes	Yes	Yes
Delplancke TDJ et al, 2018	Yes	Yes	No	Yes	Yes	Unclear	Yes	Yes
Luthra G et al, 2018	Yes	Yes	No	No	Yes	Yes	Yes	Yes
Gong S et al, 2018	No	Yes	No	No	Yes	Unclear	Yes	Yes
Morillon AC et al, 2018	Yes	Yes	No	Yes	Yes	Yes	Yes	Yes

For peer review only

For peer review only - http://bmjopen.bmj.com/site/about/guidelines.xhtml

Studies	Applicability concerns		
	**Patient selection**	**Index test**	**Reference standard**
	Are there concerns that the included patients do not match the review question?	Are there concerns that the index test, its conduct, or interpretation differ from the review question?	Are there concerns that the target condition as defined by the reference standard does not match the review question?
Grandone E et al, 2006	No	No	Yes
van Eijsden M et al, 2008	No	No	No
Horgan R et al, 2011	No	No	No
Costet N et al, 2012	No	No	No
Ertl R et al, 2012	No	No	No
Gernand AD et al, 2013	No	No	No
Sulek K et al, 2014	No	No	Yes
Choi R et al, 2016	Unclear	No	No
Kiely ME et al, 2016	No	No	No
Ong YL et al, 2016	No	No	No
Wang Y et al, 2016	No	No	No
Delplancke TDJ et al, 2018	No	Unclear	No
Luthra G et al, 2018	No	No	No
Gong S et al, 2018	Yes	Yes	No
Morillon AC et al, 2018	No	No	No

For peer review only

PRISMA 2009 Checklist

Examining the predictive accuracy of metabolomics for small for gestational age babies: a systematic review

Debora F. B. Leite & Aude-Claire Morillon, Elias F. Melo Júnior, Renato T. Souza, Fergus P. McCarthy, Ali S. Khashan, Philip N. Baker, Louise C. Kenny, José Guilherme Cecatti

Section/topic	#	Checklist item	Reported on page #
**TITLE**			
Title	1	Identify the report as a systematic review, meta-analysis, or both.	1
**ABSTRACT**			
Structured summary	2	Provide a structured summary including, as applicable: background; objectives; data sources; study eligibility criteria, participants, and interventions; study appraisal and synthesis methods; results; limitations; conclusions and implications of key findings; systematic review registration number.	3-4
**INTRODUCTION**			
Rationale	3	Describe the rationale for the review in the context of what is already known.	5
Objectives	4	Provide an explicit statement of questions being addressed with reference to participants, interventions, comparisons, outcomes, and study design (PICOS).	6
**METHODS**			
Protocol and registration	5	Indicate if a review protocol exists, if and where it can be accessed (e.g., Web address), and, if available, provide registration information including registration number.	6
Eligibility criteria	6	Specify study characteristics (e.g., PICOS, length of follow-up) and report characteristics (e.g., years considered, language, publication status) used as criteria for eligibility, giving rationale.	7-8
Information sources	7	Describe all information sources (e.g., databases with dates of coverage, contact with study authors to identify additional studies) in the search and date last searched.	6-7/ 9
Search	8	Present full electronic search strategy for at least one database, including any limits used, such that it could be repeated.	6-7
Study selection	9	State the process for selecting studies (i.e., screening, eligibility, included in systematic review, and, if applicable, included in the meta-analysis).	7-8
Data collection process	10	Describe method of data extraction from reports (e.g., piloted forms, independently, in duplicate) and any processes for obtaining and confirming data from investigators.	8
Data items	11	List and define all variables for which data were sought (e.g., PICOS, funding sources) and any assumptions and simplifications made.	7-8
Risk of bias in individual studies	12	Describe methods used for assessing risk of bias of individual studies (including specification of whether this was done at the study or outcome level), and how this information is to be used in any data synthesis.	8-9

For peer review only - http://bmjopen.bmj.com/site/about/guidelines.xhtml

PRISMA 2009 Checklist

Examining the predictive accuracy of metabolomics for small for gestational age babies: a systematic review

Debora F. B. Leite & Aude-Claire Morillon, Elias F. Melo Júnior, Renato T. Souza, Fergus P. McCarthy, Ali S. Khashan, Philip N. Baker, Louise C. Kenny, José Guilherme Cecatti

Summary measures	13	State the principal summary measures (e.g., risk ratio, difference in means).	9
Synthesis of results	14	Describe the methods of handling data and combining results of studies, if done, including measures of consistency (e.g., I^2) for each meta-analysis.	9

Section/topic	#	Checklist item	Reported on page #
Risk of bias across studies	15	Specify any assessment of risk of bias that may affect the cumulative evidence (e.g., publication bias, selective reporting within studies).	8-9
Additional analyses	16	Describe methods of additional analyses (e.g., sensitivity or subgroup analyses, meta-regression), if done, indicating which were pre-specified.	7
**RESULTS**			
Study selection	17	Give numbers of studies screened, assessed for eligibility, and included in the review, with reasons for exclusions at each stage, ideally with a flow diagram.	9
Study characteristics	18	For each study, present characteristics for which data were extracted (e.g., study size, PICOS, follow-up period) and provide the citations.	9-13
Risk of bias within studies	19	Present data on risk of bias of each study and, if available, any outcome level assessment (see item 12).	23-24
Results of individual studies	20	For all outcomes considered (benefits or harms), present, for each study: (a) simple summary data for each intervention group (b) effect estimates and confidence intervals, ideally with a forest plot.	20-22
Synthesis of results	21	Present results of each meta-analysis done, including confidence intervals and measures of consistency.	24
Risk of bias across studies	22	Present results of any assessment of risk of bias across studies (see Item 15).	9; 23-24
Additional analysis	23	Give results of additional analyses, if done (e.g., sensitivity or subgroup analyses, meta-regression [see Item 16]).	NA
**DISCUSSION**			
Summary of evidence	24	Summarize the main findings including the strength of evidence for each main outcome; consider their relevance to key groups (e.g., healthcare providers, users, and policy makers).	24-28
Limitations	25	Discuss limitations at study and outcome level (e.g., risk of bias), and at review-level (e.g., incomplete retrieval of identified research, reporting bias).	28-29
Conclusions	26	Provide a general interpretation of the results in the context of other evidence, and implications for future research.	29-30
**FUNDING**			

For peer review only

For peer review only - http://bmjopen.bmj.com/site/about/guidelines.xhtml

PRISMA 2009 Checklist

Examining the predictive accuracy of metabolomics for small for gestational age babies: a systematic review

Debora F. B. Leite & Aude-Claire Morillon, Elias F. Melo Júnior, Renato T. Souza, Fergus P. McCarthy, Ali S. Khashan, Philip N. Baker, Louise C. Kenny, José Guilherme Cecatti

Funding	27	Describe sources of funding for the systematic review and other support (e.g., supply of data); role of funders for the systematic review.	38-39

From: Moher D, Liberati A, Tetzlaff J, Altman DG, The PRISMA Group (2009). Preferred Reporting Items for Systematic Reviews and Meta-Analyses: The PRISMA Statement. PLoS Med 6(7): e1000097. doi:10.1371/journal.pmed1000097

For more information, visit: www.prisma-statement.org.

Page 2 of 2

For peer review only

BMJ Open

Examining the predictive accuracy of metabolomics for small for gestational age babies: a systematic review

Journal:	*BMJ Open*
Manuscript ID	bmjopen-2019-031238.R1
Article Type:	Research
Date Submitted by the Author:	13-Jul-2019
Complete List of Authors:	Leite, Debora; Campinas' State University, Department of Tocogynecology; Universidade Federal de Pernambuco,<br>Morillon, Aude-Claire; University College Cork National University of Ireland, Irish Centre for Fetal and Neonatal Translational Research (INFANT)<br>Melo Júnior, Elias; Universidade Federal de Pernambuco, Clinics Hospital<br>Souza, Renato; Universidade Estadual de Campinas, Obstetrics and Gynecology;<br>McCarthy, F. P.; St Thomas Hosp<br>Khashan, Ali; University College Cork, Department of Epidemiology and Public Health<br>Baker, Philip ; University of Leicester, College of Medicine<br>Kenny, Louise; University of Liverpool School of Life Sciences, Department of Women's and Children's Health<br>Cecatti, Jose; University of Campinas, Obstetrics and Gynecology
**Primary Subject Heading**:	Obstetrics and gynaecology
Secondary Subject Heading:	Epidemiology
Keywords:	small for gestational age, fetal growth restriction, metabolomics, gas-chromatography, mass spectrometry

SCHOLARONE™
Manuscripts

RESEARCH ARTICLE: SYSTEMATIC REVIEW

Examining the predictive accuracy of metabolomics for small for gestational age babies: a systematic review

Debora F. B. LEITE[a,b,c1] (0000-0001-8839-3934; deborafariasleite@gmail.com) and Aude-Claire MORILLON[d1] (0000-0002-9692-9396; audeclaire.morillon@ucc.ie); Elias F. de MELO JUNIOR[a,b] (0000-0001-7897-8090; eliasmelojr@gmail.com); Renato Teixeira SOUZA[c] (0000-000209075-9269; renatotsouzasp@gmail.com); Fergus P McCARTHY[d] (0000-0001-5062-6851; fergus.mccarthy@ucc.ie); Ali S. KHASHAN[d,e] (0000-0002-7499-9406; a.khashan@ucc.ie); Philip N. BAKER[f] (0000-0002-4592-6427; philip.baker@leicester.ac.uk); Louise C. KENNY[g] (0000-0002-9011-759X; louise.kenny@liverpool.ac.uk); Jose G. CECATTI[c] (0000-0003-1285-8445; cecatti@unicamp.br).

[a]Federal University of Pernambuco, Pernambuco, Brazil.

[b]Clinics Hospital of Federal University of Pernambuco, Recife, Pernambuco, Brazil

[c]Department of Gynecology and Obstetrics, University of Campinas, Campinas, Sao Paulo, Brazil.

[d]The Irish Centre for Fetal and Neonatal Translational Research (INFANT), University College Cork, Cork, Co. Cork, Ireland.

[e]School of Public Health, University College Cork, Cork, Co. Cork, Ireland.

[f]College of Life Sciences, University of Leicester, United Kingdom.

[g]Faculty of Health and Life Sciences, Department of Women's and Children's Health, Institute of Translational Medicine, University of Liverpool, United Kingdom.

For peer review only

[1]These two authors contributed equally.

CORRESPONDING AUTHOR

José Guilherme Cecatti

Rua Alexander Fleming, 101 Cidade Universitaria Zeferino Vaz.

13083-889. Campinas, Sao Paulo, Brazil.

Telephone: +55 19-35219482

Email: cecatti@unicamp.br

Word Count:

Abstract: 289 words

Main text 4,167 words

For peer review only

ABSTRACT

Introduction: To date, there is no robust enough test to predict small for gestational age (SGA) infants, which are at increased life-long risk of morbidity and mortality.

Objective: To determine the accuracy of metabolomics in predicting SGA babies and elucidate which metabolites are predictive of this condition.

Data sources: Two independent researchers explored 11 electronic databases and grey literature in February 2018 and November 2018, covering publications from 1998 to 2018. Both researchers performed data extraction and quality assessment independently. A third researcher resolved discrepancies.

Study eligibility criteria: Cohort or nested case-control studies were included, which investigated pregnant women and performed metabolomics analysis to evaluate SGA infants. The primary outcome was birthweight <10th centile - as a surrogate for fetal growth restriction - by population-based or customized charts.

Study appraisal and synthesis methods: Two independent researchers extracted data on study design, obstetric variables and sampling, metabolomics technique, chemical class of metabolites, and prediction accuracy measures. Authors were contacted to provide additional data when necessary.

Results: A total of 9,181 references were retrieved. Of these, 273 were duplicate, 8,760 were removed by title or abstract, and 133 were excluded by full text content. Thus, 15 studies were included. Only two studies used the 5th centile as a cutoff, and most reports sampled 2nd trimester pregnant women. Liquid-chromatography coupled to mass spectrometry was the most common metabolomics approach. Untargeted studies in the 2nd trimester provided the largest number of predictive metabolites, using maternal blood or hair. Fatty acids, phosphosphingolipids, and amino acids were the most prevalent predictive chemical subclasses.

For peer review only

Conclusions and Implications: Significant heterogeneity of participant characteristics and methods employed among studies precluded a meta-analysis. Compounds related to lipid metabolism should be validated up to the 2nd trimester in different settings.

Systematic review registration number: CRD42018089985.

Keywords: small for gestational age, fetal growth restriction, metabolomics, prediction, gas-chromatography, mass spectrometry, vitamin D, homocysteine, lipids, fatty acids.

STRENGHTS AND LIMITATIONS OF THIS STUDY

- To our knowledge, this is the first systematic review to assess the predictive accuracy of metabolomics for an adverse pregnancy outcome.
- Using SGA as surrogate for fetal growth restriction – just as in epidemiological investigations – improves the translational potential of metabolomics.
- Identification of techniques, types of maternal samples and chemical classes paves the way for future metabolomics investigations on fetal growth patterns.
- Available data could not support a meta-analysis; further studies should include accuracy measures of individual metabolites or chemical subclasses in predicting SGA.

ORIGINAL PROTOCOL: Leite DFB, Morillon A-C, Melo Júnior EF, *et al*. Metabolomics for predicting fetal growth restriction: protocol for a systematic review and meta-analysis. *BMJ Open* 2018;8:e022743. doi:10.1136/bmjopen-2018-022743.

For peer review only

INTRODUCTION

Fetal growth restriction (FGR) and small for gestational age (SGA) infants are major concerns in modern obstetrics. [1–3] SGA is commonly used as a proxy for FGR, [4] despite the subtle differences between these two pathological conditions. The prevalence of both varies according to criteria applied and on the population and setting, although it reaches as much as 25% in low and middle-income countries. [5] SGA newborns may have adverse health effects, such as stillbirth, [4] perinatal asphyxia, [6] impaired neurodevelopment, [7] and increased cardiovascular risk. [8,9] To date, there are no robust prediction tools for SGA using clinical factors, [10,11] ultrasound data, [12,13] or placental biomarkers. [14]

For hypothesis generating or validation purposes, metabolomics is a novel area of biomarker, discovery, development and clinical diagnostics in translational medicine. [15,16] Metabolomics is the study of all metabolites [15,16] in a given sample, i.e. low molecular weight compounds (50-2000 Da) that are intermediates of biochemical reactions and metabolic pathways, considered to directly reflect cellular activity and phenotype. [15,16] Recent studies have evaluated the pathophysiology [17–20] of SGA with metabolomics. However, little is known about the potential of metabolomics to identify predictive compounds of SGA.

Since metabolomics can identify multiple metabolites from low volume samples, and create a model from a collection of these samples, [15] it is a promising technology for hypothesis generation in a heterogeneous condition such as SGA. The prediction of SGA in pregnancy would help refer women to specialized care facilities, improving maternal and neonatal outcomes. [21,22]

In this context, our review question was “What is the accuracy of metabolomics for predicting FGR?”. Then, the main objective of this systematic

For peer review only

review was to assess the accuracy of metabolomics techniques in predicting SGA. As a secondary aim, we intended to determine which metabolites are predictive of this condition.

METHODS

The protocol for this systematic review was published previously. [23] This study follows international guidelines for transparency (PROSPERO, CRD 42018089985) and respects the Preferred Reporting Items for Systematic Reviews and Meta-Analysis (PRISMA) statement. [24] For this systematic review, ethical approval was unnecessary.

Literature Search Strategy

Two independent researchers (DFBL and ACM) assessed 11 electronic databases (PubMed, EMBASE, Latin American and Caribbean Health Sciences Literature (LILACS), Scientific Electronic Library Online (Scielo), Health Technology Assessment (HTA), Database of Abstracts of Reviews of Effects (DARE), Aggressive Research Intelligence Facility (ARIF), Cumulative Index of Nursing and Allied Health Literature (CINAHL), Maternity and Infant Care (MIDIRS), Scopus, and Web of Science) and grey literature. There were no limits or language constraints; the search strategy covered published documents between 1998 and 2018. Keywords 'small for gestational age', 'metabolomics', 'prediction', 'antenatal', and variations of each, were combined with Boolean operators depending on each database requirements. The full EMBASE literature search was, as follows: ('fetal growth retardation' OR 'fetal growth restriction' OR 'intrauterine growth restriction' OR 'intrauterine growth retardation' OR 'small for gestational age') AND ('metabolomic*' OR 'metabonomic*'

For peer review only

OR ‘metabolit* ‘H NMR’ OR ‘proton NMR’ OR ‘proton nuclear magnetic resonance’ OR ‘liquid chromatogra*’ OR ‘gas chromatogra*’ OR ‘UPLC’ OR ‘ultra-performance’ OR ‘ultra performance liquid chromatograph*’) AND (‘pregnan*’ OR ‘antenat*’ OR ‘ante nat*’ OR ‘prenat*’ OR ‘pre nat*’) AND (‘screen*’ OR ‘predict*’ OR ‘metabolic profil*’). Please check Supplementary Material 1 for more details.

Outcomes and subgroup analysis

The primary outcome was SGA, as a surrogate for FGR and defined as birthweight <10th centile, by population-based or customized charts. Secondary outcomes were birthweight ≤5th or ≤3rd centile.

The intended subgroup analysis comprised: type of metabolomics technique applied (nuclear magnetic resonance, NMR; gas or liquid chromatography coupled with mass spectrometry, GC-MS or LC-MS respectively); maternal health status before pregnancy (women with *versus* without any chronic health condition); type of SGA suspected during pregnancy (early *versus* late SGA); and type of pregnancy (singleton *versus* multiple pregnancy).

Selection Criteria of Studies, Data Collection and Analysis

Cohort or case-control studies were included if maternal samples were collected before the clinical diagnosis of SGA, if any metabolomics technique was applied, and if the results of SGA were presented. Articles presenting data from the same research project but analyzing distinct metabolites or showing data from different countries were included. Studies were excluded (i) according to study design; (ii) if they had not applied any metabolomics technique; (iii) if they were only experimental studies; (iv) if it was not possible to extract data on SGA; (v) or if they presented

For peer review only

duplicate data, in which case the most complete publication was included for final analysis.

Two researchers (DFBL and ACM) independently selected studies, extracted data and discussed discrepancies. One additional reviewer (EFMJ or RTS) helped to decide, by majority, when no consensus was reached.

Piloted standardized forms were applied for data extraction, including pregnancy characteristics and experimental details. The Human Metabolome Database (HMDB) [25] and the Kyoto Encyclopedia of Genes and Genomes [26] were used for matching chemical class and metabolic pathways of each metabolite, respectively.

Risk of bias and Assessment of concerns regarding applicability

Two researchers (DFBL and ACM) independently evaluated individual studies using the QUADAS-2 tool. [27] One of the third reviewers (EFMJ, or RTS) helped in decision-making when no consensus was achieved.

Each study was classified as high, low, or unclear risk of bias in four Domains (Patient Selection, Index Test, Reference Standard, and Flow and Timing), and as high, low, or unclear concerns regarding applicability in the first three Domains. We did not consider two signaling questions ("Was a case-control design avoided?", "Was there an appropriate interval between the index test and reference standard?"). The nested case-control design was an inclusion criterion and maternal samples should have been collected during pregnancy, i.e. before the SGA diagnosis. Studies were considered 'low risk', for example, (i) if pregnancy or neonatal complications were not excluded in just one group of participants or data on participant selection had been provided; (ii) if methods for sample preparation and

For peer review only - http://bmjopen.bmj.com/site/about/guidelines.xhtml

interpretation were standardized or metabolite threshold was defined before the experiments (for targeted analysis); (iii) if the adequacy and reasons for choosing the reference birthweight chart had been explained; or, (iv) if large for gestational age babies had been excluded from the final comparative analysis.

Data synthesis

A quantitative summary of data was performed when any predictive accuracy measures could be extracted. Authors were contacted to provide additional information, when necessary. However, only Delplancke et al [28] replied. The estimation of likelihood ratios and hierarchical summary receiver operator characteristic curve [29] were planned, as well as assessment of heterogeneity and publication bias. [30] However, due to lack of data, a meta-analysis could not be performed.

Patient and Public Involvement

There was no patient or public involvement in conducting this systematic review.

Data Availability Statement

All data relevant to this systematic review are included in this manuscript - in the article or uploaded as supplementary information. There are no individual patient identifiable data.

RESULTS

Literature search characteristics

For peer review only

The literature search for this systematic review was performed in February 2018, and re-run in November 2018. A total of 9,181 references were retrieved (Figure 1). After the removal of duplicate records (n=273), title and abstract screening, and analysis of the remaining 148 full-text articles, 15 articles were included. [17,18,28,31–42] See Supplementary Material 2 for excluded studies.

Characteristics of the included studies

The characteristics of the included studies are shown in Table 1. The prevalence of SGA ranged from 7.3% [33] to 21.5% in cohort studies. [28] There were no studies using a birthweight ≤3rd centile for a definition of SGA. The time interval between initial participant enrollment and publication varied from three [17] to 54 years, [40] although these data were unclear in 38% of the reports. [18,28,32,33,37] In nested case-control studies, participants were matched by maternal age, [17,18,38,42] ethnicity, [17,18,42] parity, [38] body mass index, [17,18,42] or infant gender. [18,38]

Participant characteristics varied between studies. Regarding gestational age at assessment, samples were collected in the 2nd trimester in one half of the studies. [17,18,33,35,37,39,42] In three reports, women were assessed at least twice. [34,38,41] In one study, maternal blood was drawn either in the 1st or 2nd trimester; [40] and in another three studies, only samples from the 3rd trimester were considered. [28,36,41] In the latter case, maternal hair was divided according to length, allowing evaluation of 2nd and 3rd trimester metabolites. [28] Studies considering the 5th centile as the cutoff, sampled women in the 1st trimester. [31,32] Twin pregnancy was a clear exclusion criterion in most studies. [17,18,31,33–35,37,40–42] Pregnancy aided by assisted reproduction [18,37] or women with pre-existing conditions [17,18,35,37,42] were also excluded, although these data were

For peer review only

incompletely reported. [28,32,36,38,39,41] When both nulliparous and multiparous women were enrolled, there was no data analysis according to parity. Half of the studies considered term deliveries exclusively, [18,28,36,38–41] and the remaining studies did not differentiate results according to gestational age at birth.

Regarding clinical risk factors for SGA, only one paper mentioned a previous history of SGA, but findings were not adjusted for this variable. [32] All studies, except one, [28] cited participant smoking status. The rate of smoking habit ranged from 2.4% [18] to 47.5%. [40] It is important to note that Gernand et al [40] analyzed samples from women recruited between 1959 and 1965, when smoking while pregnant was encouraged, which explains the high rate of smoking participants. The duration of smoking or any differences in birthweight (absolute measures or centiles) were not clearly stated. Although more prevalent in SGA pregnancies, results did not change with this variable control. [31,32,35,37,40] Only Gong et al [41] mentioned the suspicion of SGA in pregnancy, exhibiting decreasing abdominal circumference growth velocity between 20-36 wks. However, on final analysis, these babies were grouped with infants not suspected during pregnancy.

For peer review only

Table 1. Main characteristics of included studies

Authors, year	Country, year of participants enrolment	Study design	Affected/ non-affected	Gestational age at assessment	Type of pregnancy	Parity	Birthweight curve
**Outcome: SGA <5th centile**							
Costet N et al, 2011	France, 2002-2006 (PELAGIE Cohort)	Nested case-control	134/ 399	11w	Single pregnancy	Nulliparous and parous women, unclear proportions	Customized curve
Ertl R et al, 2012	United Kingdom [a]	Nested case-control	150/ 1,000	11^{+0}-13^{+6}w	Unclear	55,3% nulliparous in SGA group, 48.1% nulliparous in control group	Population-based charts
**Outcome: SGA <10th centile**							
Grandone E et al, 2006	Italy [a]	Cohort	31/ 393	17.1 ± 1.2w[b] (mean)	Single pregnancy; no maternal pre-existing conditions	Unclear	Population-based charts
van Eijsden M et al, 2008	Netherlands, 2003-2004 (ABCD Study)	Cohort	429/ 3275	13.5 ± 3.3w (mean)	Term deliveries, no diabetes or hypertension	57.6% nulliparous	Population-based charts
Horgan RP et al, 2011	Australia, 2008-2011 (SCOPE Cohort)	Nested case-control	40/ 40	14-16w	Single pregnancy; no other pregnancy complications	Nulliparous	Customized curve
Gernand AD et al, 2013	United States, 1959-1965 (Collaborative Perinatal Project)	Nested case-control	395/ 1751	≤26w	Single pregnancy; term deliveries	Parous women	Population-based charts
Sulek K et al, 2014	Singapore [a] (GUSTO Study)	Nested case-control	41/ 42	26-28w	Single pregnancy; term deliveries; no maternal pre-existing conditions	Nulliparous and parous women, unclear proportions	Population-based charts
Choi R et al, 2016	South Korea, 2012-2013	Cohort	39/ 217	1st, 2nd or 3rd trimester	Single pregnancies	Nulliparous and parous women, unclear	Population-based charts

For peer review only

For peer review only - http://bmjopen.bmj.com/site/about/guidelines.xhtml

						proportions	
Kiely ME et al, 2016	Ireland, 2008-2011 (SCOPE Cohort)	Cohort	190/ 1578	14-16w	Single pregnancy; no maternal pre-existing conditions	Nulliparous	Customized curve
Ong YL et al, 2016	Singapore [a] (GUSTO Study)	Cohort	83/ 827	26-28w	Single pregnancy; no maternal chronic illness	43,5% nulliparous	Population-based charts
Wang Y et al, 2016	Taiwan, 2000-2001 (Taiwan Maternal and Infant Cohort Study)	Cohort	35/ 188	3rd trimester	Unclear; term deliveries	48% nulliparous	Population-based charts
Delplancke TDJ et al, 2018	New Zealand [a]	Cohort	20/ 73	34-37w	Unclear; term deliveries	Unclear	Customized curve
Luthra G et al, 2018	United States, 2010-2012 (TIDES Study)	Nested case-control	53/ 106	1st and 2nd trimester	Single pregnancies; term deliveries	60% nulliparous	Customized curve
Gong S et al, 2018	United Kingdom, 2008-2012 (POP study)	Nested case-control	162/259	36w	Single pregnancies; term deliveries	Nulliparous	Customized curve
Morillon A-C et al, 2018	2008-2011 (SCOPE Study)	Nested case-control	40/40	20w	Single pregnancies	Nulliparous	Customized curve

[a] Unclear period of participant recruitment. [b] Mean for all study participants.

For peer review only

Subgroup analysis

Due to unavailable data, the only subgroup analysis performed was related to the metabolomics approach applied (Table 2). There was no mention of adherence to metabolomics reporting data guidelines. LC-MS was the leading technique used. Three studies have investigated metabolites related to environmental exposure, from contaminated water, [31] consumer products,[36] or pesticides, [42] while others have analyzed endogenous compounds. [32–35,37–40] Only Luthra et al conducted a biomarker validation study, [38] while Gong et al [41] chose to analyze the top ten statistically different metabolites according to infant sex.

Maternal blood was the most common biological sample analyzed by LC-MS in all studies, [17,32,34–37,39–41] except for one, which used GC-MS.[39] Maternal urine was analyzed by NMR, [38] GC-MS [36] or LC-MS. [42] There was only one report using amniotic fluid [33] and two using maternal hair, [18,28] all applying GC-MS. The period of laboratory analysis was rarely specified, which made it impossible to estimate total time of sample storage.

Untargeted studies reported diverse metabolic features. Authors matched the peaks with an in-house library [18,28] or HMDB-related database. [17,42] Horgan et al [17] found 785 compounds both in maternal and newborn samples; their predictive model included 19 metabolites (only five could be putatively identified, Table 2) and used 2nd trimester maternal blood. Sulek et al [18] and Delplancke et al [28] prepared and analyzed samples with GC-MS using similar protocols. Sulek et al [18] identified 32 statistically different chromatographic features from which they built a predictive model using five metabolites, including two fatty acids (2-methyloctadecanoate and margarate). In contrast, Delplancke et al, [28] identified 198 metabolites, including three fatty acids (margaric, pentadecanoic, and myristic

For peer review only

acid) showing significantly higher levels in SGA cases, when 2nd trimester maternal hair segments were studied.

For peer review only

Table 2. Subgroup analysis of included studies according to which metabolomics technique was applied.

Authors/ year	Metabolomics Technique	Maternal sample/ Storage temperature	Prediction model*	Targeted compounds	Coefficient of variation/ Limits of quantitation	Predictive compounds	Sensitivity/ Specificity	AUC
**Nuclear magnetic resonance**								
Luthra G et al, 2018	^{1}H-NMR 1D NOESY with pre-saturation and homonuclear 2D *J*-resolved at 300 K<br>Bruker 600 MHz Advance III HD spectrometer	Urine/ -80°C	Targeted	Tyrosine, acetate, formate, trimethylamine	NA	None		
**Gas chromatography coupled to mass spectrometry**								
Costet N et al, 2011	GC-MS<br>Simple head space SPME-Capillary GC	Urine/ -20°C	Targeted	Trichloroacetic acid	<5%/ 0.01mg/L	None	0.1/ 0.93	
Sulek K et al, 2014	GC-MS<br>Thermo Trace GC Ultra system coupled to ISQ mass selective detector<br>Capillary GC column: Phenomenex ZB-1701 (30 m x 250 µm id x 0.15 µm with 5 m guard column)	Hair/ -20°C	Untargeted	NA	NA	↓ Lactate<br>↓ Levulinate<br>↑2-methyloctadecanate<br>↑Tyrosine<br>↓ Margarate		0.998
Delplancke TDJ et al, 2018	GC-MS:<br>Agilent 7890B gas chromatograph, capillary column ZB-1701 (30m x 250µm id x 0.15µm with 5m guard column)<br>5977 A mass spectrometer, electron impact ionisation	Hair/ -20°C	Untargeted	NA	NA	↑ Margaric acid<br>↑ Pentadecanoic acid<br>↑ Myristic acid[c]		0.72<br>0.73<br>0.73

For peer review only

**Liquid chromatography coupled to mass spectrometry**								
Grandone E et al, 2006	LC-MS/MS triple quadrupole Applera API 3000, TurboIonSpray ionisation	Amniotic fluid/ -80ºC	Targeted	Homocysteine	Unclear	↑Homocysteine (1,29µM; 1,05-1,51µM)		
Horgan RP et al, 2011	UPLC- MS/MS Thermo Fisher LTQ Orbitrap, ESI	Plasma/ -80ºC	Untargeted	NA	NA	Hexacosanedioic acid, diglyceride, lyso-phosphocholine, sphinganine 1-phosphate; sphingosine 1-phosphate[d]		0.90
Ertl R et al, 2012	HPLC- MS/MS Shimadzu Prominence HPLC system with a column Phenomenex Luna C8 3 x 50 mm; AbSciex API-5000 triple quadrupole, ESI	Serum/ -80ºC	Targeted	25(OH)D_2; 25(OH)D_3	6.3%[a], 6.6%[b] (D_2); 6.5%[a], 7.3%[b] (D_3)/ unclear	↓25,OH,Vitamin D (12.16ng/mL; 8.09-20.54ng/mL)	0.72/ 0.45	
Gernand AD et al, 2013	LC-MS/MS	Serum/ -20ºC	Targeted	25(OH)D_2; 25(OH)D_3	8.2%[a] (D_2) 5.9%[a] (D_3)/ <1ng/mL	None	0.39/ 0.66	
Choi R et al, 2016	HPLC- MS/MS Waters HPLC system, Applied Biosystems API-4000 MS/MS mass spectrometer	Serum/ -20ºC	Targeted	Methylmalonic acid; homocysteine	<10%[a]; <10%[b]/ Unclear	None		
Kiely ME et al, 2016	UPLC- MS/MS Waters Acquity UPLS system, Waters Triple Quadrupole TQD mass spectrometer	Serum/ -80ºC	Targeted	25(OH)D_2; 25(OH)D_3; 3-epi-25(OH)D_3.	<6%[a]; <5%[b]/ 0.57ng/mL (D_2); 0.26ng/mL (D_3), 0,41ng/mL (epi-D_3)	None		

For peer review only

Ong YL et al, 2016	LC-MS/MS Applied Biosystems ThermoHypersil BDS C8 reverse-phase column	Plasma/ Unclear	Targeted	$25(OH)D_2$; $25(OH)D_3$	≤10,3%[a,b]/ <1,6ng/mL	None	0.12/ 0.87
Wang Y et al, 2016	LC-MS Agilent HPLC system, Applied Biosystems Sciex API-4000 triple quadrupole mass spectrometer	Serum/ Unclear	Targeted	PFOA; long-chain PFCA	0,83-7,94%[a]; 1,57-24,7%[b]/ 0,07-0,45ng/mL[e]	PFDeA (OR 3,14; 95%CI 1,07-9,19), PFUnDA (OR 1,83; 95%CI 1,01-3,32)[f]	
Gong S et al, 2018	LC-MS/MS Shimadzu UK Limited UPLC system, ACE Excel 2 C18-PFP LC-column; Thermo Fisher Scientific Exactive orbitrap mass spectrometer	Serum/ Unclear	Untargeted	NA		↑N1,N12-diacetylspermine[f]	
Morillon A-C et al, 2018	UPLC- MS/MS Waters Acquity UPLS system, Waters Synapt G2-S mass spectrometer	Urine/-80oC	Untargeted	NA		None	
Others							
van Eijsden M et al, 2008	GC-FID Solid phase extraction SPE, Capillary GC	Plasma/ -80°C	Semi-targeted, Lipid extraction	Elaidic, linoleic, alfa-linolenic, eicosatetraenoic, EPA, DPA, DHA DGLA, AA, Adrenic, and Osbond acids	≤2 - 22%[b]/ Unclear	↓ Eicosatetraenoic acid (OR 1,5; 95%CI 1,07-2,11), ↓DPA (OR 1,49; 95% CI 1,06-2,1)	

[a]Intra-assay and [b]inter-assay coefficients of variation. [c]These metabolites were found in 2nd trimester hair segments. [d]And more 14 metabolites that could not be identified certain based on chromatographic peak and mass: Phenylacetylglutamine or formyl-N-acetyl-5-methroxykynurenamine; leucyl-leucyl-norleucine or sphingosine 1-phosphate; cervonyl carnitine and/or 1-alpha,25-dihydroxy-18-oxocholecalciferol; (15Z)-tetracosenoic acid or 10,13-dimethyl-11-docosyne-10,13-diol or trans-selacholeic acid; pencosenoic acid or cyclohexyl acetate or octanoic acid or methyl-heptenoic acid or 4-hydroxy-2-octenal or DL-2-aminooctanoic acid or 3-amino-octanoic acid; hydroxybutyrate or hydroxy-methylpropanoate or methyl methoxyacetate; lysophosphocoline and phosphocoline (more than 10 hits); phosphocoline (more than 20 hits); phosphocoline or ubiquinone-8; acetylleucil-leucil-norleucinal or oleoylglycerone phosphate or LPA(0:0/18:2(9Z,12Z)) or 1-16:1lysoPE or phosphocoline(O-11:1(10E)/2:0) or (3s)-3,4-Di-N-hexanoyloxybutyl-1-phosphocoline or N-(3-hydroxy-propyl) arachidonoyl amine or N-methyl N-(2-hydroxy-ethyl) arachidonoyl amine or similar; lysophosphocholine (16:1) or cervonyl carnitine; preganediol-3-glucuronide or 3-alpha,20-alpha-dihydroxy-5-beta-pregnane-3-glucuronide; 6-hydroxyshingosine or

For peer review only

(4OH,8Z,t18:1) sphingosine or 15-methyl-15-prostaglandin D2 or 15-R-prostaglandin E2 methylester. [e]Values for all studied metabolites. [f]Predictive compounds only for female babies.

AUC: area under the receiver operating characteristic curve; ^{1}H-NMR: hydrogen nuclear magnetic resonance; NOESY: nuclear Overhauser effect spectroscopy; GC-MS: gas chromatography coupled to mass spectrometry; SPME: solid phase micro extraction; LC-MS: liquid chromatography coupled to mass spectrometry; UPLC: ultra-performance liquid chromatography; ESI: Electrospray ionisation; FID: flame ionisation detection; PFOA: perfluorooctanoic acid; PFCA: perfluorocarboxylic acid; PFDeA: perfluorodecanoic acid; PFUnDA: perfluoroundecanoic acid; EPA: eicoisapentaenoic acid; DPA: docosapentaenoic acid; DHA: docosahexaenoic acid; DGLA: dihomo-gama-linolenic acid; AA: arachidonic acid; OR: odds ratio; CI: confidence interval; NA: not applicable.

For peer review only

Analysis of identified metabolites

The identified compounds refer to eleven HMDB chemical classes. Fatty acids [18,28,39] comprised the most prevalent chemical class, followed by amino acids [18,33] and phosphosphingolipids [17] (Table 3).

A total of 5,974 women were assessed for vitamin D status. Results were presented as total vitamin D, [32,35,37,40] although vitamin D_2, D_3 or 3-epi-25(OH)D_3 [35] metabolites were measured. Results were stratified according to season of maternal sampling or latitude. Either <15ng/mL (<37.5nmol/L) [40] or <20ng/mL (<50nmol/L) [32,35,37] levels characterized vitamin D deficiency, but were statistically different in SGA pregnancies only in the 1st trimester. [32] Horgan et al found a metabolite that could represent a vitamin D derivative, but it was only predictive in combination with 18 other compounds; this model had an area under the curve (AUC) of 0.90 (optimal odds ratio (OR), 44; 95%CI 9-214). [17]

The second most frequent targeted metabolite was homocysteine, [33,34] although levels were only differentiated between normal and SGA pregnancies when measured in 2nd trimester amniotic fluid, with a multiple linear regression model r^2=0.012 and p=0.029. [33] Comparatively, the only common metabolite in 2nd trimester maternal hair was margarate, with conflicting results since it was found to be either increased (AUC 0.72, 95%CI 0.58-0.86) [28] or decreased. [18] The N1,N12-diacetylspermine and the perfluorocarboxylic acids were associated to female SGA babies, not males. The former presented a 5-fold decreased risk of SGA across quintiles. The perfluorodecanoic and perfluoroundecanoic acids presented OR of 3.14 (95%CI 1.07-9.19) and 1.83 (95%CI 1.01-3.32). [36] Tyrosine, an essential amino acid for infants, was part of the predictive model of maternal hair, combining 5 metabolites with an AUC of 0.998 (95%CI 0.992-1.0) [18]. However,

For peer review only

tyrosine did not predict SGA when urine samples were studied. [38] Methylmalonic acid, [34] acetate, formate, or trimethylamine, [38] did not differentiate SGA when compared to uncomplicated pregnancies (p>0.05).

For peer review only

Table 3. Predictive metabolites summarized according to their chemical class, subclass, and biological process

Predictive metabolites	Chemical class	Chemical subclass	Metabolic pathway
Margarate	Fatty acyls	Fatty acids and conjugates	Lipid transport, metabolism, peroxidation
Pentadecanoic acid	Fatty acyls	Fatty acids and conjugates	Lipid transport, metabolism, peroxidation; fatty acid metabolism and biosynthesis
Myristic acid	Fatty acyls	Fatty acids and conjugates	Lipid transport, metabolism, peroxidation; fatty acid metabolism and biosynthesis
Eicosatetraenoic acid	Fatty acyls	Fatty acids and conjugates	Lipid transport, metabolism, peroxidation; lipid metabolism pathway
Docosapentaenoic acid	Fatty acyls	Fatty acids and conjugates	Lipid transport and metabolism, fatty acid metabolism, alpha linolenic acid and linoleic acid metabolisms
Tyrosine [a]	Carboxylic acids and derivatives	Amino acids, peptides, and analogues	Catecholamine biosynthesis; phenylalanine and tyrosine metabolism; thyroid hormone synthesis; transcription and translation
Homocysteine	Carboxylic acids and derivatives	Amino-acids, peptides, and analogues	Glycine and serine metabolism; methionine metabolism
Hexacosanedioic acid	Carboxylic acids and derivatives	Dicarboxylic acid and derivatives	Fatty acid biosynthesis
Sphinganine 1-phosphate	Sphingolipids	Phosphosphingolipids	Sphingolipid signalling pathway, nneuroactive ligand-receptor interaction
Sphingosine 1-phosphate	Sphingolipids	Phosphosphingolipids	Lipid metabolism pathway, sphingolipid metabolism
PFDeA	Alkyl halides	Alkyl fluorides	Not reported[b]
PFUnDA	Alkyl halides	Alkyl fluorides	Not reported[b]
25,OH,Vitamin D	Steroids and steroids derivatives	Vitamin D and derivatives	Lipid metabolism pathway
Diglyceride	Glycerolipids	Diradylglycerols	Adipocytokine signaling pathway
Lactate	Hydroxy acids and derivatives	Alpha hydroxy acids and derivatives	Gluconeogenesis, glycogenosis types IB and IC, pyruvate metabolism, triosephosphate isomerase
N1,N12-diacetylspermine	Carboximidic acids and derivatives	Carboximidic acids	
Lyso-phosphocholine	Glycerophospholipids	Glycerophosphocholines	Not reported[b]
2-methyloctadecanate	Saturated hydrocarbons	Alkanes	Not reported[b]
Levulinate	Keto acids and derivatives	Gamma-keto acids and derivatives	Not reported[b]

[a] Essential amino acid for infants. [b] No human metabolic pathways reported at KEGG. PFDeA: perfluorodecanoic acid; PFUnDA: perfluoroundecanoic acid.

For peer review only

Risk of bias and Applicability Concerns

Figure 2 shows synthesized data for all included studies. See Supplementary Material 3 for individual QUADAS-2 data.

Regarding the risk of bias, all cohort studies conducted a consecutive participant inclusion. [28,33–37,39] Nested case-controls matched cases and controls randomly [33–35,41] or according to maternal and infant characteristics. [17,18,38,42] One study [41] failed to mention matching procedures ('Patient Selection' domain). Researchers were not blinded to SGA status when interpreting metabolomics results, [17,18,28,32,35–41] and thresholds of targeted metabolites were not pre-specified [31,33,36,38,39] ('Index Test' domain). Conversely, SGA identification was not influenced by the metabolomics test, although it was unclear when laboratory experiments were performed in some studies. [18,28,31,33,34,41] Birthweight charts were adequate, except for two studies. The first did not report which centile was chosen, [18] and the second used a centile designed for a different population [33] ('Reference Test' domain). Two studies were ranked as 'high risk' because not all participants were included in the analysis [31,37] ('Flow and Timing' domain).

The QUADAS-2 tool also highlights the importance of how the findings of the included studies are suitable to the review question. In the Patient Selection domain, it was ranked as 'high applicability concerns' when infants born between the 4th and the 10th centile, but with normal abdominal circumference growth velocity, were not included in final analysis. [41] It was 'unclear' when the gestational age of maternal assessment was not standardized, [34] or was inferred by hair segment length; [28] or when few metabolites from untargeted studies were chosen for interpretation [41]

For peer review only - http://bmjopen.bmj.com/site/about/guidelines.xhtml

(‘Index Test’ domain). Finally, it was ‘high’ when the birthweight charts applied did not correspond to the study population [18,33] (‘Reference Standard’ domain).

Meta-analysis

From the 15 included studies, only three were designed for prediction purposes [17,18,42] and provided the AUC. The remaining reports described statistical differences of metabolites between SGA pregnancies and controls. [28,31–41] Accuracy measures were extracted when available (Table 2). However, due to marked heterogeneity (Tables 1 and 2) of gestational age at sampling, type of samples used, type of birthweight chart chosen, thresholds for vitamin D deficiency, metabolomics approach, and identified compounds, a meta-analysis could not be performed.

DISCUSSION

Main findings

In this first systematic review of metabolomics and adverse pregnancy endpoints, we presented techniques and metabolites, which were studied for the prediction of SGA. Any effect on birthweight has important implications for perinatal research, since it is related to short and long-term outcomes, [43–46] and in different generations. [47,48] Intrauterine environment influences fetal growth through epigenetic processes: altered gene expression potentially leads to distinct phenotypes. [49] Metabolomics is the most adequate approach to study this outcome, since it is most directly related to phenotype. [50]

Interpretation of metabolomics findings in pregnancy can be challenging. Firstly, maternal metabolites concentrations are influenced by placental transfer to

For peer review only

and from the fetus. The 'mirror effect', seen for maternal plasma and venous cord blood metabolites at birth, [51] cannot be ruled out when only maternal specimens are studied. Secondly, maternal exposure to distinct compounds may affect metabolite levels. Statistically significant differences between SGA infants and controls may not express the totality of underlying pathological pathways and have no clinical meaning. Finally, it is unclear when the processes leading to SGA are initiated. The disruption in maternal metabolism can theoretically occur at any time. In general the lower the gestational age at which the condition is suspected, the more severe the phenotype will be at birth. [52,53] Thus, the description of clinical data in translational studies must deal with all these confounding factors.

Gestational age at sampling is probably the most important parameter for prediction purposes. With timely prediction, women could be referred to specialized care, have increased surveillance, and this in turn may lead to a reduction in perinatal mortality. There are temporal changes in the maternal metabolome during pregnancy; [28,54–57] therefore, it is reasonable to expect distinctive metabolites at different stages of pregnancy, as reported here. Unfortunately, a wide or unclear definition of gestational age of sampling [34,36,38,40] render a more precise interpretation impossible, and may limit the clinical application of these results.

In contrast, gestational age at birth and birthweight centile seem to be the hallmarks of severity and prognosis of growth restriction. [6,58] Indeed, term and preterm SGA babies show distinct clinical phenotypes, and there are concerns that some babies <10th centile of birthweight are constitutionally small infants. [59–61] If only term deliveries are evaluated, the most severe cases of growth restriction may be potentially missed. Moreover, when term and preterm births are analyzed together, or when lower cutoffs are not specified (e.g. ≤3rd or ≤5th centile), the lack of

For peer review only

predictive metabolites might mean that they are distinct conditions. Thus, we hypothesize that the predictive performance of metabolomics may be improved if data is analyzed by gestational age at delivery, and by different cutoffs of birthweight centiles.

Evidence suggests that tobacco smoke has an impact on birthweight, [62–64] although it is uncertain how and when fetal growth is impaired. It is possibly related to oxidative stress, [65] and both maternal and fetal metabolism may be disturbed at delivery. [66,67] Studies that were included did not investigate cigarette-related chemicals or quantify exposure to tobacco smoke. Therefore, no relationship between SGA and tobacco was found. Hence, we suggest that tobacco interferes with ongoing metabolic pathological processes, or its disturbance is related to additional metabolic pathways other than the one examined by the included studies.

Subgroup and metabolite findings

No reports have explored data on any maternal chronic condition, suspicion of SGA in pregnancy, or number of fetuses. The lack of clear statements about participant selection have hindered data interpretation and precluded these analyses.

The majority of included studies performed a targeted approach, i.e. a hypothesis-testing evaluation, [16,50] driven by epidemiological or experimental data regarding SGA newborns. None of the targeted metabolites [31–40] were in common with those found by 'hypothesis-generating' metabolic profiling [17,18,28,41,42] investigations. This reinforces the suggestion that various maternal metabolic pathways may be triggered by the SGA condition, and be detected by different biological samples. However, since blood is a very complex sample and GC-MS only

For peer review only

evaluates volatile molecules, [50] therefore our findings may be biased by study methodologies.

Untargeted studies, as expected, have characterized several metabolites that may be validated in future investigations. Nine lipids and fatty acid metabolites, [17,18,28,39] two amino acids, [18,33] and a steroid [17,32] have been identified as potential biomarkers of SGA.

All lipid-related metabolites identified are intermediates for energy storage and breakdown. Most metabolites were found in maternal blood [17] or hair of the SGA group. [18,28] Blood levels of saturated and monounsaturated non-esterified fatty acids apparently remain stable throughout pregnancy, while long chain polyunsaturated fatty acid (DHA and EPA, for example) measurements seem to show ethnicity-related changes. [57] Experimental data shows the importance of hypoxia and oxidative stress to placental function and ultimately, to birthweight. [68,69] Findings from included studies may represent a dysregulation of lipid pathways at the placental level, but an association with maternal background is unclear. Therefore, we hypothesize that disorders of lipid metabolism may be the 'metabolic snapshot' of defective deep placentation, [70] and might reflect maternal efforts to respond to impaired fetal growth.

Recommendations on the assessment of vitamin D and cutoffs to define vitamin D deficiency in pregnancy are controversial. [71] However, vitamin D supplementation decreases SGA risk. [72] In early pregnancy, vitamin D status has been related to SGA, [73,74] which is in accordance with this review, despite the inconsistent findings. [75] There is evidence that trophoblasts actively produce and secrete vitamin D metabolites, [76] but it is not clear how they mediate fetal growth impairment. Altered hepatic gene expression and liver function in vitamin D deficient

For peer review only

female rats, [77] and single nucleotide polymorphisms [78] in vitamin D receptor gene have been suggested as mechanisms to be explored by a multidimensional omics approach.

Finally, homocysteine is an intermediate metabolite of the folate cycle. It is indirectly involved with DNA methylation and is a marker of folate deficiency. [79] Maternal levels rarely reach hyperhomocysteinemia limits, [80] but folate depletion [81–83] and homocysteine itself[80] are thought to be associated with a higher SGA risk. In this review, homocysteine was only statistically different in SGA pregnancies when measured in amniotic fluid, [33] although within the normal ranges proposed for 17-21 weeks. [84] Since amniocentesis is generally performed in women at higher obstetrical risk, future studies should investigate whether homocysteine in amniotic fluid represents a confounding factor or a new biomarker. [85]

Methodological quality

Most studies were ranked as ‘low risk’ of bias or applicability to the review question. However, the lack of clear descriptions of laboratory experiments, including sample preparation and storage, and blinding of the researchers to the case/control status, are major pitfalls of the included studies.

Strengths and limitations

To our knowledge, this is the first systematic review of metabolomics and an adverse pregnancy outcome (SGA). We presented possible biomarkers of SGA pathophysiology, metabolites implicated in lipid transport and metabolic pathways, as well as gluconeogenesis.

For peer review only

However, this analysis has some limitations. First, included studies showed heterogeneity, which is fundamental in systematic reviews. Indeed, there was a wide variety of participant characteristics and methods used, and not all authors provided a detailed description of methods employed. Although the Metabolomics Standard Initiative was released in 2007, [86] there is still poor adherence to guidelines. [87,88] Clear reporting [15,87,88] and data sharing in repositories are crucial steps in identifying features of interest, specifically possible biomarkers to be validated in the clinical studies. [15] Secondly, we could not perform a meta-analysis of the extracted data, impacting the translational potential of metabolomics.

Thirdly, we considered that birthweight was a surrogate measure of intrauterine development. SGA and FGR are not interchangeable concepts. However, SGA has been used as a surrogate for FGR in many clinical studies due to difficulties in defining optimal intrauterine growth: (i) FGR diagnosis relies mostly on ultrasound measurements of fetal biometry, [3,89] which in turn is subject to systematic errors; [90] (ii) intrauterine development is adaptive, rather than uniform [91] or only genetically driven; [49] (iii) growth impairment at birth better identifies adverse neonatal outcomes than during pregnancy. [58] It is recognized that changes in obstetric care occur when growth restriction is suspected, and neonatal outcomes are improved. [21,22] Thus, an accurate prediction of SGA during pregnancy will be a turning point in modern obstetrics.

CONCLUSIONS AND IMPLICATIONS FOR PRACTICE

Using the available clinical tools, efforts to predict SGA remain disappointing. Since SGA is a heterogeneous condition, it benefits from metabolomics. This novel area of research allows analysis of numerous types of biological fluids and detects

For peer review only

thousands of metabolites in complex samples. [15,16,25] However, findings of this systematic review must be interpreted with caution. The type of samples used may have influenced LC-MS (2nd trimester maternal blood) and GC-MS (2nd trimester maternal hair) findings in individual studies. Furthermore, the prediction of SGA in the context of maternal disorders, suspected FGR and twin pregnancies is an open field for future metabolomics studies, and environmental exposure investigation as well.

Surprisingly, none of the studies used ≤3rd centile of birthweight as a cutoff or analyzed preterm deliveries and hypertensive syndromes. Considering our findings and the different phenotypic manifestations of SGA, we envision a better performance when (i) cutoffs other than the 10th centile are tested; (ii) data on gestational age at sampling and at birth are standardized; and (iii) other pregnancy-related syndromes are considered, especially hypertension. Thus, future metabolomics results should advance in these critical points.

Finally, all detected biomarkers were related to lipid pathways and energy metabolism. We consider that research efforts to predict SGA should focus on compounds involved in these pathways, up to the 2nd trimester of pregnancy.

For peer review only

REFERENCES

1 American College of Obstetricians and Gynecologists (ACOG). Practice Bulletin 134. Fetal Growth Restriction. *Obstet Gynecol* 2013;**121**:1122–33.

2 Figueras F, Gratacós E. Update on the diagnosis and classification of fetal growth restriction and proposal of a stage-based management protocol. *Fetal Diagn Ther* 2014;**36**:86–98. doi:10.1159/000357592

3 Gordijn SJ, Beune IM, Thilaganathan B, *et al.* Consensus definition of fetal growth restriction: a Delphi procedure. *Ultrasound Obstet Gynecol* 2016;**48**:333–9. doi:10.1002/uog.15884

4 Bukowski R, Hansen NI, Willinger M, *et al.* Fetal Growth and Risk of Stillbirth: A Population-Based Case-Control Study. *PLoS Med* 2014;**11**. doi:10.1371/journal.pmed.1001633

5 Lee ACC, Katz J, Blencowe H, *et al.* National and regional estimates of term and preterm babies born small for gestational age in 138 low-income and middle-income countries in 2010. *Lancet Glob Heal* 2013;**1**:e26-36. doi:10.1016/S2214-109X(13)70006-8

6 Mendez-Figueroa H, Truong VTT, Pedroza C, *et al.* Small-for-gestational-age infants among uncomplicated pregnancies at term: a secondary analysis of 9 Maternal-Fetal Medicine Units Network studies. *Am J Obstet Gynecol* 2016;**215**:628.e1-628.e7. doi:10.1016/j.ajog.2016.06.043

7 Sharma D, Farahbakhsh N, Shastri S, *et al.* Intrauterine growth restriction–part 2. *J Matern Neonatal Med* 2016;**29**:4037–48. doi:10.3109/14767058.2016.1154525

8 Barker DP, Osmond C, Simmonds SJ, *et al.* The relation of small head circumference and thinness at birth to death from cardiovascular disease in adult life. *Br Med J* 1993;**306**:422–6. doi:10.1136/bmj.306.6875.422

9 Balasuriya CND, Stunes AK, Mosti MP, *et al.* Metabolic Outcomes in Adults Born Preterm with Very Low Birth Weight or Small for Gestational Age at Term: A Cohort Study. *J Clin Endocrinol Metab* 2018;**103**:4437–46. doi:10.1210/jc.2018-00464

10 Goto E. Maternal anthropometry to predict small for gestational age: A meta-analysis. *Eur J Obstet Gynecol Reprod Biol* 2016;**203**:193–8. doi:10.1016/j.ejogrb.2016.05.042

11 Pay ASD, Frøen JF, Staff AC, *et al.* Prediction of small-for-gestational-age status by symphysis–fundus height: a registry-based population cohort study. *BJOG* 2016;**123**:1167–73. doi:10.1111/1471-0528.13727

12 Parry S, Sciscione A, Haas DM, *et al.* Role of early second-trimester uterine artery Doppler screening to predict small-for-gestational-age babies in nulliparous women. *Am J Obstet Gynecol* 2017;**217**:594.e1-594.e10. doi:10.1016/j.ajog.2017.06.013

13 Roma E, Arnau A, Berdala R, *et al.* Ultrasound screening for fetal growth restriction at 36 vs 32 weeks' gestation: A randomized trial (ROUTE). *Ultrasound Obstet Gynecol* 2015;**46**:391–7. doi:10.1002/uog.14915

14 Conde-Agudelo A, Papageorghiou a T, Kennedy SH, *et al.* Novel biomarkers for predicting intrauterine growth restriction: a systematic review and meta-analysis. *BJOG* 2013;**120**:681–94. doi:10.1111/1471-0528.12172

15 Xia J, Broadhurst DI, Wilson M, *et al.* Translational biomarker discovery in clinical metabolomics: An introductory tutorial. *Metabolomics* 2013;**9**:280–99. doi:10.1007/s11306-012-0482-9

16 Patti GJ, Yanes O, Siuzdak G. Metabolomics: The apogee of the omics trilogy. *Nat Rev Mol cell Biol* 2012;**13**:263–9. doi:10.1038/nrm3314

17 Horgan RP, Broadhurst DI, Walsh SK, *et al.* Metabolic profiling uncovers a phenotypic signature of small for gestational age in early pregnancy. *J Proteome Res* 2011;**10**:3660–73.

18 Sulek K, Han TL, Villas-Boas SG ranat., *et al.* Hair metabolomics: identification of fetal compromise provides proof of concept for biomarker discovery. *Theranostics* 2014;**4**:953–9. doi:10.7150/thno.9265

19 Favretto D, Cosmi E, Ragazzi E, *et al.* Cord blood metabolomic profiling in intrauterine growth restriction. *Anal Bioanal Chem* 2012;**402**:1109–21. doi:10.1007/s00216-011-5540-z

20 Sanz-Cortés M, Carbajo RJ, Crispi F, *et al.* Metabolomic profile of umbilical cord blood plasma from early and late intrauterine growth restricted (IUGR) neonates with and without signs of brain vasodilation. *PLoS One* 2013;**8**:e80121. doi:10.1371/journal.pone.0080121

21 Monier I, Blondel B, Ego A, *et al.* Does the Presence of Risk Factors for Fetal Growth Restriction Increase the Probability of Antenatal Detection? A French National Study. *Paediatr Perinat Epidemiol* 2016;**30**:46–55. doi:10.1111/ppe.12251

22 Verlijsdonk JW, Winkens B, Boers K, *et al.* Suspected versus non-suspected small-for-gestational age fetuses at term: Perinatal outcomes. *J Matern Neonatal Med* 2012;**25**:938–43. doi:10.3109/14767058.2011.600793

23 Leite DFB, Morillon A-C, Melo Júnior EF, *et al.* Metabolomics for predicting fetal growth restriction: protocol for a systematic review and meta-analysis. *BMJ Open* 2018;**8**:e022743. doi:10.1136/bmjopen-2018-022743

24 Moher D, Liberati A, Tetzlaff J, *et al.* Preferred Reporting Items for Systematic Reviews and Meta-Analyses: the PRISMA statement. *Ann Intern Med* 2009;**151**:264–9. doi:10.1371/journal.pmed1000097

25 Wishart DS, Feunang YD, Marcu A, *et al.* HMDB 4.0: The human metabolome database for 2018. *Nucleic Acids Res* 2018;**46**:D608–17. doi:10.1093/nar/gkx1089

26 Kyoto Encyclopedia of Genes and Genomes. https://www.genome.jp/kegg/ (accessed 20 Dec 2018).

27 Whiting PF, Rutjes AWS, Westwood ME, *et al.* Research and Reporting Methods Accuracy Studies. *Ann Intern Med* 2011;**155**:529–36. doi:10.7326/0003-4819-155-8-201110180-00009

28 Delplancke TDJ, De Seymour J V., Tong C, *et al.* Analysis of sequential hair segments reflects changes in the metabolome across the trimesters of pregnancy. *Sci Rep* 2018;**8**:1–12. doi:10.1038/s41598-017-18317-7

29 Rutter CM, Gatsonis CA. A hierarchical regression approach to meta-analysis of diagnostic test accuracy evaluations. *Stat Med* 2001;**20**:2865–84. doi:10.1002/sim.942

30 Deeks JJ, Macaskill P, Irwig L. The performance of tests of publication bias and other sample size effects in systematic reviews of diagnostic test accuracy was assessed. *J Clin Epidemiol* 2005;**58**:882–93. doi:10.1016/j.jclinepi.2005.01.016

31 Costet N, Garlantézec R, Monfort C, *et al.* Environmental and urinary markers of prenatal exposure to drinking water disinfection by-products, fetal growth, and duration of gestation in the PELAGIE birth cohort (Brittany, France, 2002-2006). *Am J Epidemiol* 2012;**175**:263–75. doi:10.1093/aje/kwr419

For peer review only

32 Ertl R, Yu CKH, Samaha R, *et al.* Maternal serum vitamin D at 11-13 weeks in pregnancies delivering small for gestational age neonates. *Fetal Diagn Ther* 2012;**31**:103–8. doi:10.1159/000333810

33 Grandone E, Colaizzo D, Vecchione G, *et al.* Homocysteine levels in amniotic fuild. *Thromb Haemost* 2006;**95**:625–8. doi:10.1160/TH05

34 Choi R, Choi S, Lim Y, *et al.* A prospective study on serum methylmalonic acid and homocysteine in pregnant women. *Nutrients* 2016;**8**:1–15. doi:10.3390/nu8120797

35 Kiely ME, Zhang JY, Kinsella M, *et al.* Vitamin D status is associated with uteroplacental dysfunction indicated by pre-eclampsia and small-for-gestational-age birth in a large prospective pregnancy cohort in Ireland with low Vitamin D status. *Am J Clin Nutr* 2016;**104**:354–61. doi:10.3945/ajcn.116.130419

36 Wang Y, Adgent M, Su PH, *et al.* Prenatal exposure to perfluorocarboxylic acids (PFCAs) and fetal and postnatal growth in the Taiwan maternal and infant cohort study. *Environ Health Perspect* 2016;**124**:1794–800. doi:10.1289/ehp.1509998

37 Ong YL, Quah PL, Tint MT, *et al.* The association of maternal Vitamin D status with infant birth outcomes, postnatal growth and adiposity in the first 2 years of life in a multi-ethnic Asian population: The Growing Up in Singapore Towards healthy Outcomes (GUSTO) cohort study. *Br J Nutr* 2016;**116**:621–31. doi:10.1017/S0007114516000623

38 Luthra G, Vuckovic I, Bangdiwala A, *et al.* First and second trimester urinary metabolic profiles and fetal growth restriction: An exploratory nested case-control study within the infant development and environment study. *BMC Pregnancy Childbirth* 2018;**18**:1–8. doi:10.1186/s12884-018-1674-8

39 van Eijsden M, Hornstra G, van der Wal MF, *et al.* Maternal n-3, n-6, and trans fatty acid profile early in pregnancy and term birth weight: a prospective cohort study. *Am J Clin Nutr* 2008;**87**:887–95.

40 Gernand AD, Simhan HN, Klebanoff MA, *et al.* Maternal serum 25-hydroxyvitamin D and measures of newborn and placental weight in a U.S. multicenter cohort study. *J Clin Endocrinol Metab* 2013;**98**:398–404. doi:10.1210/jc.2012-3275

41 Gong S, Sovio U, Aye ILMH, *et al.* Placental polyamine metabolism differs by fetal sex, fetal growth restriction, and preeclampsia. *JCI Insight* 2018;**3**:1–15. doi:10.1172/jci.insight.120723

42 Morillon A-C, Yakkundi S, Thomas G, *et al.* Untargeted UPLC-MS analysis of potential pesticide and biomarkers of fetal growth restriction. *Conf Proceedings 14th Annu Conf Metabolomics Soc* 2018;:200.

43 Wang N, Wang X, Li Q, *et al.* The famine exposure in early life and metabolic syndrome in adulthood. *Clin Nutr* 2017;**36**:253–9. doi:10.1016/j.clnu.2015.11.010

44 Hales CN, Barker DJP. The thrifty phenotype hypothesis. *Br Med Bull* 2001;**60**:5–20. doi:10.1093/bmb/60.1.5

45 Melo AS, Vieira CS, Barbieri MA, *et al.* High prevalence of polycystic ovary syndrome in women born small for gestational age. *Hum Reprod* 2010;**25**:2124–31. doi:10.1093/humrep/deq162

46 Ravelli a CJ, Vandermeulen JHP, Michels RPJ, *et al.* Glucose-tolerance in adults after prenatal exposure to famine. *Lancet* 1998;**351**:173–7. doi:10.1016/S0140-6736(97)07244-9

For peer review only

47 Chamorro-Garcia R, Diaz-Castillo C, Shoucri BM, *et al.* Ancestral perinatal obesogen exposure results in a transgenerational thrifty phenotype in mice. *Nat Commun* 2017;**8**. doi:10.1038/s41467-017-01944-z

48 Seferovic MD, Goodspeed DM, Chu DM, *et al.* Heritable IUGR and adult metabolic syndrome are reversible and associated with alterations in the metabolome following dietary supplementation of 1-carbon intermediates. *FASEB J* 2015;**29**:2640–52. doi:10.1096/fj.14-266387

49 Padmanabhan V, Cardoso RC, Puttabyatappa M. Developmental programming, a pathway to disease. *Endocrinology* 2016;**157**:1328–40. doi:10.1210/en.2016-1003

50 Dunn WB, Broadhurst DI, Atherton HJ, *et al.* Systems level studies of mammalian metabolomes: The roles of mass spectrometry and nuclear magnetic resonance spectroscopy. *Chem Soc Rev* 2011;**40**:387–426. doi:10.1039/b906712b

51 Visentin S, Crotti S, Donazzolo E, *et al.* Medium chain fatty acids in intrauterine growth restricted and small for gestational age pregnancies. *Metabolomics* 2017;**13**:1–9. doi:10.1007/s11306-017-1197-8

52 Korzeniewski SJ, Allred EN, Joseph RM, *et al.* Neurodevelopment at age 10 years of children born <28 weeks with fetal growth restriction. *Pediatrics* 2017;**140 (5) (n**. doi:http://dx.doi.org/10.1542/peds.2017-0697

53 Lees C, Marlow N, Arabin B, *et al.* Perinatal morbidity and mortality in early-onset fetal growth restriction: Cohort outcomes of the trial of randomized umbilical and fetal flow in Europe (TRUFFLE). *Ultrasound Obstet Gynecol* 2013;**42**:400–8. doi:10.1002/uog.13190

54 Luan H, Meng N, Liu P, *et al.* Non-targeted metabolomics and lipidomics LC-MS data from maternal plasma of 180 healthy pregnant women. *Gigascience* 2015;**4**:16–9. doi:10.1186/s13742-015-0054-9

55 Di Giulio AM, Carelli S, Castoldi RE, *et al.* Plasma amino acid concentrations throughout normal pregnancy and early stages of intrauterine growth restricted pregnancy. *J Matern Neonatal Med* 2004;**15**:356–62. doi:10.1080/14767050410001725578

56 Orczyk-Pawilowicz M, Jawien E, Deja S, *et al.* Metabolomics of human amniotic fluid and maternal plasma during normal pregnancy. *PLoS One* 2016;**11**:1–13. doi:10.1371/journal.pone.0152740

57 Lindsay KL, Hellmuth C, Uhl O, *et al.* Longitudinal metabolomic profiling of amino acids and lipids across healthy pregnancy. *PLoS One* 2015;**10**:1–19. doi:10.1371/journal.pone.0145794

58 Boghossian NS, Geraci M, Edwards EM, *et al.* Neonatal and fetal growth charts to identify preterm infants <30 weeks gestation at risk of adverse outcomes. *Am J Obstet Gynecol* 2018;**219**:195.e1-195.e14. doi:10.1016/j.ajog.2018.05.002

59 Katz J, Wu LA, Mullany LC, *et al.* Prevalence of small-for-gestational-age and its mortality risk varies by choice of birth-weight-for-gestation reference population. *PLoS One* 2014;**9**:1–9. doi:10.1371/journal.pone.0092074

60 Ray JG, Jiang D, Sgro M, *et al.* Thresholds for Small for Gestational Age Among Newborns of East Asian and South Asian Ancestry. *J Obstet Gynaecol Canada* 2009;**31**:322–30. doi:10.1016/S1701-2163(16)34149-4

61 Cheng YKY, Leung TY, Lao TTH, *et al.* Impact of replacing Chinese ethnicity-specific fetal biometry charts with the INTERGROWTH-21ststandard. *BJOG An Int J Obstet Gynaecol* 2016;**123**:48–55. doi:10.1111/1471-0528.14008

For peer review only

62 Abraham M, Alramadhan S, Iniguez C, *et al.* A systematic review of maternal smoking during pregnancy and fetal measurements with meta-analysis. *PLoS One* 2017;**12**:1–13. doi:10.1371/journal.pone.0170946

63 Vesterinen HM, Morello-Frosch R, Sen S, *et al.* Cumulative effects of prenatal-exposure to exogenous chemicals and psychosocial stress on fetal growth: Systematic-review of the human and animal evidence. *PLoS One* 2017;**12**. doi:10.1371/journal.pone.0176331

64 Dessì A, Corona L, Pintus R, *et al.* Exposure to tobacco smoke and low birth weight: from epidemiology to metabolomics. *Expert Rev Proteomics* 2018;**15**:647–56. doi:10.1080/14789450.2018.1505508

65 Stone WL, Bailey B, Khaisha N. The pathophysiology of smoking during pregnancy: a systems biology approach. *Front Biosci* 2014;**E6**:318–28.

66 Fischera T, Lilic LN, Lic S, *et al.* Low-level maternal exposure to nicotine associates with significant metabolic perturbations in second-trimester amniotic fluid. *Env Int* 2017;**107**:227–34. doi:10.1001/jamasurg.2014.1086.Feasibility

67 Rolle-Kampczyk UE, Krumsiek J, Otto W, *et al.* Metabolomics reveals effects of maternal smoking on endogenous metabolites from lipid metabolism in cord blood of newborns. *Metabolomics* 2016;**12**. doi:10.1007/s11306-016-0983-z

68 Thomas MM, Haghiac M, Grozav C, *et al.* Oxidative Stress Impairs Fatty Acid Oxidation and Mitochondrial Function in the Term Placenta. *Reprod Sci* 2018;:193371911880205. doi:10.1177/1933719118802054

69 Määttä J, Sissala N, Dimova EY, *et al.* Hypoxia causes reductions in birth weight by altering maternal glucose and lipid metabolism. *Sci Rep* 2018;**8**:13583. doi:10.1038/s41598-018-31908-2

70 Brosens I, Pijnenborg R, Vercruysse L, *et al.* The 'great obstetrical syndromes' are associated with disorders of deep placentation. *Am J Obs Gynecol* 2011;**204**:193–201. doi:10.1016/j.ajog.2010.08.009.THE

71 Holick MF, Binkley NC, Bischoff-Ferrari HA, *et al.* Evaluation, treatment, and prevention of vitamin D deficiency: An endocrine society clinical practice guideline. *J Clin Endocrinol Metab* 2011;**96**:1911–30. doi:10.1210/jc.2011-0385

72 Bi WG, Nuyt AM, Weiler H, *et al.* Association between Vitamin D supplementation during pregnancy and offspring growth, morbidity, and mortality: A systematic review and meta-analysis. *JAMA Pediatr* 2018;**172**:635–45. doi:10.1001/jamapediatrics.2018.0302

73 Bodnar LM, Catov JM, Zmuda JM, *et al.* Maternal Serum 25-Hydroxyvitamin D Concentrations Are Associated with Small-for-Gestational Age Births in White Women. *J Nutr* 2010;**140**:999–1006. doi:10.3945/jn.109.119636.pregnancy

74 Leffelaar ER, Vrijkotte TGM, Van Eijsden M. Maternal early pregnancy vitamin D status in relation to fetal and neonatal growth: Results of the multi-ethnic Amsterdam Born Children and their Development cohort. *Br J Nutr* 2010;**104**:108–17. doi:10.1017/S000711451000022X

75 Martínez-Domínguez SJ, Tajada M, Chedraui P, *et al.* Systematic review and meta-analysis of Spanish studies regarding the association between maternal 25-hydroxyvitamin D levels and perinatal outcomes. *Gynecol Endocrinol* 2018;:1–8. doi:10.1080/09513590.2018.1472761

76 Park H, Wood MR, Malysheva O V., *et al.* Placental Vitamin D metabolism and its associations with circulating Vitamin D metabolites in pregnant women. *Am J Clin Nutr* 2017;**106**:1439–48. doi:10.3945/ajcn.117.153429

For peer review only

77 Sharma SS, Jangale NM, Harsulkar AM, *et al. Chronic maternal calcium and 25-hydroxyvitamin D deficiency in Wistar rats programs abnormal hepatic gene expression leading to hepatic steatosis in female offspring*. 2017. doi:10.1016/j.jnutbio.2017.01.008

78 Barchitta M, Maugeri A, La Rosa MC, *et al.* Single nucleotide polymorphisms in vitamin D receptor gene affect birth weight and the risk of preterm birth: Results from the “mamma & bambino” cohort and a meta-analysis. *Nutrients* 2018;**10**:18. doi:10.3390/nu10091172

79 Kim J, Kim H, Roh H, *et al.* Causes of hyperhomocysteinemia and its pathological significance. *Arch Pharm Res* 2018;**41**:372–83. doi:10.1007/s12272-018-1016-4

80 Yajnik CS, Chandak GR, Joglekar C, *et al.* Maternal homocysteine in pregnancy and offspring birthweight: Epidemiological associations and Mendelian randomization analysis. *Int J Epidemiol* 2014;**43**:1487–97. doi:10.1093/ije/dyu132

81 Smits LJM, Essed GGM. Short interpregnancy intervals and unfavourable pregnancy outcome: Role of folate depletion. *Lancet* 2001;**358**:2074–7. doi:10.1016/S0140-6736(01)07105-7

82 van Eijsden M, van der Wal MF, Bonsel GJ. Association between short interpregnancy intervals and term birth. *Am J Clin Nutr* 2008;**88**:147–53. doi:10.1016/0013-4694(81)90034-1

83 Hogeveen M, Blom HJ, Den Heijer M. Maternal homocysteine and small-for-gestational-age offspring: Systematic review and meta-analysis. *Am J Clin Nutr* 2012;**95**:130–6. doi:10.3945/ajcn.111.016212

84 Imbard A, Blom HJ, Schlemmer D, *et al.* Methylation metabolites in amniotic fluid depend on gestational age. *Prenat Diagn* 2013;**33**:848–55. doi:10.1002/pd.4142

85 Heazell A, Newman L, Lean S, *et al.* Pregnancy outcome in mothers over the age of 35. *Curr Opin Obs Gynecol* 2018;**30**:337–43.

86 Goodacre R, Broadhurst D, Smilde AK, *et al.* Proposed minimum reporting standards for data analysis in metabolomics. *Metabolomics* 2007;**3**:231–41. doi:10.1007/s11306-007-0081-3

87 Considine EC, Thomas G, Boulesteix AL, *et al.* Critical review of reporting of the data analysis step in metabolomics. *Metabolomics* 2018;**14**. doi:10.1007/s11306-017-1299-3

88 Spicer RA, Salek R, Steinbeck C. Compliance with minimum information guidelines in public metabolomics repositories. *Sci Data* 2017;**4**:1–8. doi:10.1038/sdata.2017.137

89 McCowan LM, Figueras F, Anderson NH. Evidence-based national guidelines for the management of suspected fetal growth restriction: comparison, consensus, and controversy. *Am J Obstet Gynecol* 2018;**218**:S855–68. doi:10.1016/j.ajog.2017.12.004

90 Lappen JR, Myers SA. The systematic error in the estimation of fetal weight and the underestimation of fetal growth restriction. *Am J Obstet Gynecol* 2017;**216**:477–83. doi:10.1016/j.ajog.2017.02.013

91 Papageorghiou AT, Ohuma EO, Altman DG, *et al.* International standards for fetal growth based on serial ultrasound measurements: The Fetal Growth Longitudinal Study of the INTERGROWTH-21st Project. *Lancet* 2014;**384**:869–79. doi:10.1016/S0140-6736(14)61490-2

For peer review only

For peer review only

AUTHORS CONTRIBUTIONS

DFBL and ACM have equally contributed to this report, and both are guarantors of this review. They elaborated the protocol, searched the literature, selected studies, extracted data, assessed risk of bias, and drafted the initial manuscript. RTS and EFMJ have participated in judging inclusion of studies, interpreting data, and revising the manuscript. FM have supported data extraction and have critically examined the clinical interpretation of results. ASK has discussed the quantitative data synthesis, and supervised the report writing. PNB, LCK, and JGC have supervised and approved all steps. All authors have read and agree with this submission.

ACKNOWLEDGEMENTS

We are grateful to (i) Shauna Barret, from the Brookfield Library, University College Cork, Ireland, for her support with the literature search; (ii) Ting-Li Han, from the Department of Obstetrics, The First Affiliated Hospital of Chongqing Medical University, China, for providing additional data for this systematic review; (iii) and Luis Felipe D'Orsi, from University of Campinas, for his support with methods' issues.

FUNDING

DFBL (process number 88881.134512/2016-01) and RTS (88881.134095/2016-01) have awarded scholarships from Brazilian Federal Agency for Support and Evaluation of Graduate Education (CAPES). ACM was granted a scholarship from Science Foundation Ireland, for her doctoral thesis. PRETERM-SAMBA has granted sponsor from Brazilian National Research Council (CNPq) (Award 401636/2013-5) and from the Bill and Melinda Gates Foundation (grant OPP1107597), corresponding

For peer review only

to the research call "Grand Challenges Brazil: Reducing the burden of preterm birth", number 05/2013. This research received no specific grant from commercial or not-for-profit sectors. Our sponsors have not intervened in authors' decision to write the systematic review protocol or to submit this paper.

COMPETING INTERESTS

None to declare.

PROVENANCE AND PEER REVIEW

Not commissioned; externally peer reviewed.

Figure captions

Figure 1. PRISMA flowchart of study identification, screening and selection. From: Moher D, Liberati A, Tetzlaff J, Altman DG, The PRISMA Group (2009). Preferred Reporting Items for Systematic Reviews and Meta-Analyses: The PRISMA Statement. PLoS Med 6(7): e1000097. doi:10.1371/journal.pmed1000097. For more information, visit www.prisma-statement.org.

Figure 2. Assessment of risk of bias (A) and applicability concerns (B) of individual studies.

Supplementary material description

Supplementary material 1 – Detailed literature search strategy.

Supplementary material 2 - List of excluded studies and reasons.

Supplementary material 3 - Individual QUADAS-2 data for all 15 included studies.

For peer review only

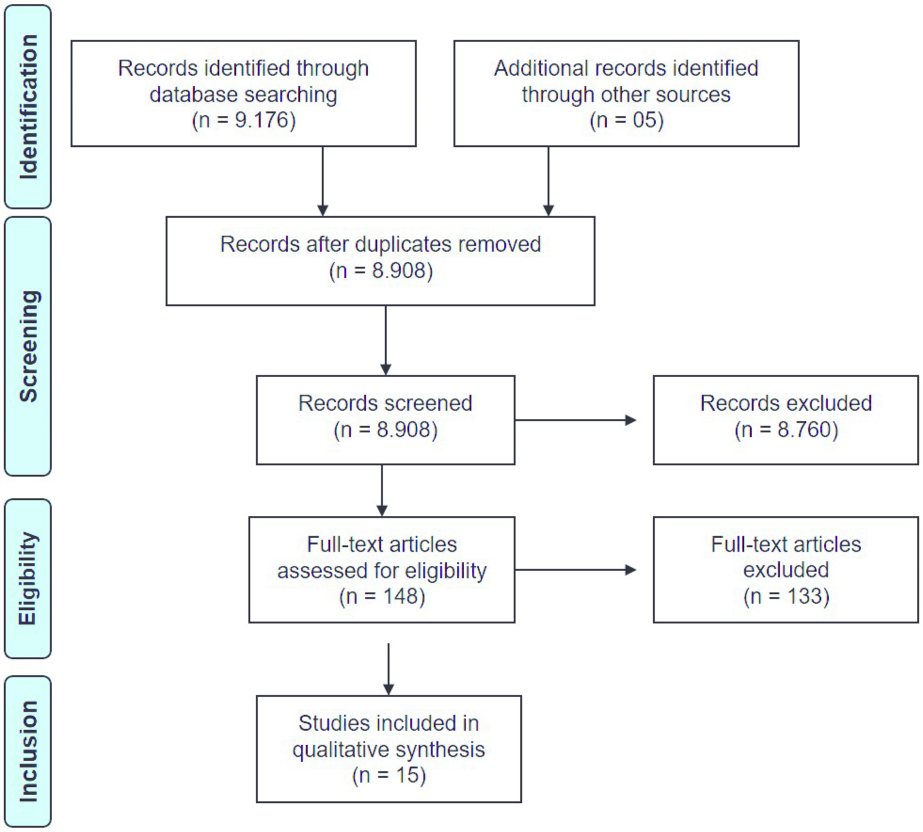

Figure 1

90x90mm (300 x 300 DPI)

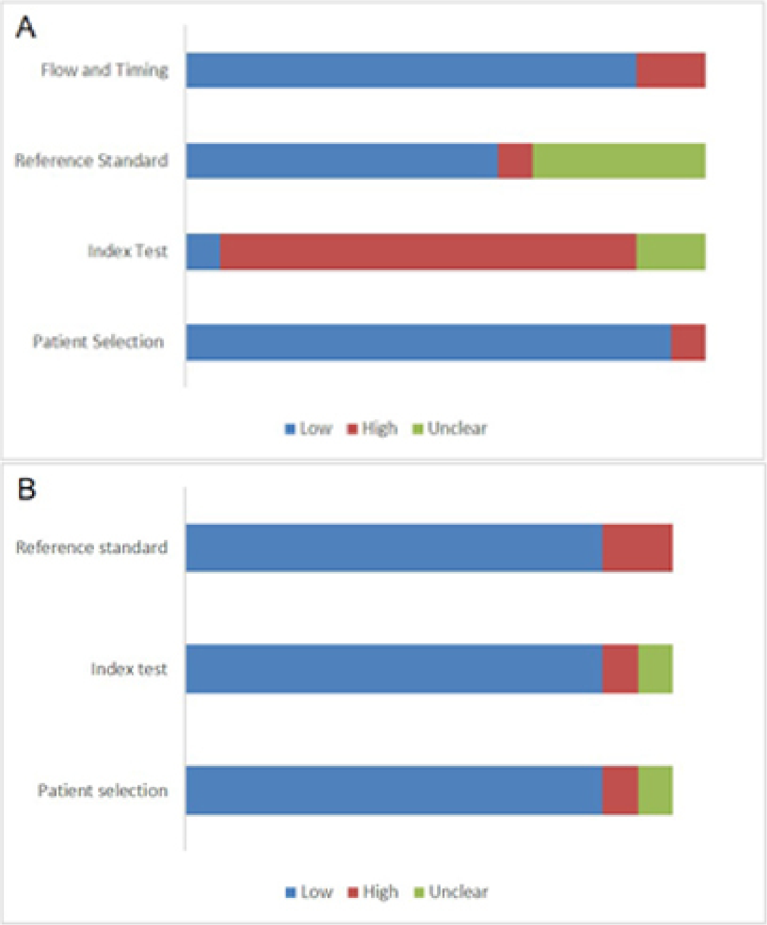

Figure 2

89x89mm (150 x 150 DPI)

Examining the predictive accuracy of metabolomics for small for gestational age babies: a systematic review

Debora F. B. Leite and Aude-Claire Morillon; Elias F. de Melo Junior; Renato Teixeira Souza; Fergus P McCarthy; Ali S. Khashan; Philip N. Baker; Louise C. Kenny; Jose G. Cecatti

Supplementary material 1 – Detailed literature search strategy.

1	fetal growth retardation
2	fetal growth restriction
3	intrauterine growth restriction
4	intrauterine growth retardation
5	small for gestational age
6	#1 OR #2 OR #3 OR #4 OR #5
7	metabolomic*
8	metabonomic*
9	metabolit*
10	H NMR
11	proton NMR
12	proton nuclear magnetic resonance
13	liquid chromatogra*
14	gas chromatogra*
15	UPLC
16	ultra-performance liquid chromatograph*
17	ultra performance liquid chromatograph*
18	#7 OR #8 OR #9 OR #10 OR #11 OR #12 OR #13 OR #14 OR #15 OR #16 OR #17
19	pregnan*
20	antenat*
21	ante nat*
22	prenat*
23	pre nat*
24	#19 OR #20 OR #21 or #22 OR #23
25	screen*
26	predict*
27	metabolic profil*
28	#25 OR #26 OR #27
29	#6 AND #18 AND #24 AND 28

For peer review only

Examining the predictive accuracy of metabolomics for small for gestational age babies: a systematic review

Debora F. B. Leite & Aude-Claire Morillon, Elias F. Melo Júnior, Renato T. Souza, Fergus P. McCarthy, Ali S. Khashan, Philip N. Baker, Louise C. Kenny, José Guilherme Cecatti.

Supplementary material 2 – List of excluded studies and reasons.

**Authors/ year**	**Country of enrollment**	**Additional comments**
Exclusions according to study design or statistical analysis		
Barnes CM et al, 2010	United States	Maternal samples collected at delivery.
Bobinski R. 2013	Poland	Cross-sectional study.
Bobinski R. 2014	Poland	Cross-sectional study.
Cao WC et al, 2016	China	Cross-sectional study. The metabolomics technique was not applied.
Chen TT et al, 2017	China	Cross-sectional study.
Cinelli et al, 2018	Italy	
D'Anna R et al, 2004	Italy	Cross-sectional study. The metabolomics technique was not applied.
Guo H et al, 2014	China	Cross-sectional study.

For peer review only

Guo J et al, 2016	China	Cross-sectional study.
Maekawa R et al, 2017	Japan	Cross-sectional study.
Mao D et al, 2010	China	Cross-sectional study.
Miranda J et al 2018	Spain	Cross-sectional study.
Powell et al, 2018	Australia	SGA babies not suspected before birth were considered healthy infants.
Spanou L. et al, 2017	Greece	Cross-sectional study.
Stein TP et al, 2008	United States	Newborns with birth defects were included in the analysis.
Tang R et al, 2013	China	Cross-sectional study.
Visentin S et al, 2017	Italy	Maternal samples collected after clinical recognition of FGR/SGA.
Zhu Y et al, 2018	China	Cross-sectional study.
Zota AR et al, 2009	United States	Cross-sectional study. The metabolomics technique was not applied.
Studies that have not applied metabolomics technique		
Baker PN, 2009	United Kingdom	
Berkowitz GS et al, 2004	United States	
Bodnar LM et al, 2012	United States	

For peer review only

Braun JM et al, 2011	United States	There is no data about FGR.
Cetin I et al, 2002	Italy	
Chong MFF et al, 2015	Singapore	There is no data about birth weight.
Colapinto CK et al, 2015	Canada	The metabolomics technique was not applied for pregnant women's specimens.
Cupul-Uicab LA et al, 2013	United States	
Fruscalzo A et al, 2015	Italy	There is no data about birth weight.
Jusko TA et al, 2006	United States	
Koepke R et al, 2004	Mexico	
Lõpez-Alarcõn M et al, 2015	Mexico	There is no data about birth weight.
Maruta E et al, 2017	Japan	
Miranda ML et al, 2015	United States	
Morley R et al, 2006	Australia	
Muthayya S et al, 2006	India	
Paşaoğlu H et al, 2003	Turkey	
Rahman A et al, 2009	Bangladesh	
Rajasingam D et al, 2009	United Kingdom	

For peer review only

Savitz DA et al, 2002	United States	The metabolomics technique was not applied for pregnant women's specimens.
Savvidou MD et al, 2003	United Kingdom	
Schneuer FJ et al, 2014	Australia	
Snijder CA et al, 2013	Netherlands	
Sweeney AM & Symanski E, 2007	United States	
Takimoto H et al, 2007	Japan	
Terrell ML et al, 2015	United States	
Wei Y et al, 2017	Bangladesh	
Weisskopf MG et al, 2005	United States	
Whyatt RM et al, 2009	United States	
Xue F et al, 2007	United States	
Studies that have not presented specific data about FGR/SGA		
Bach CC et al, 2016	Denmark	
Bachkangi P et al.	United Kingdom	
Bahado-Singh RO et al, 2012	United Kingdom	

For peer review only

Bahado-Singh RO et al, 2015	United Kingdom
Bahado-Singh RO et al, 2017	United Kingdom
Bentley-Lewis R, 2015	United States
Braun JM et al, 2009	United States
Buckley JP et al, 2016	United States
Cantonwine D et al, 2010	Mexico
Cantonwine D et al, 2015	United States
Casas M et al, 2016	Spain
Castorina R et al, 2017 (a)	United States
Chou WC et al, 2014.	Taiwan
Cunha Figueiredo AC et al, 2017	Brazil
Dalsager L et al, 2018	Denmark
De Renzy-Martin KT. et al, 2014	Poland
Debost-Legrand A et al, 2016	France
Desert et al, 2015	France

For peer review only

Diaz SO et al, 2011	Portugal
Diaz SO et al, 2013	Portugal
Dobierzewska A et al, 2017	Chile
Dudzik D et al, 2015	Spain.
Engström KS et al, 2010	Bangladesh
Ettinger AS et al, 2017	Canada
Feng L et al, 2016	China
Ferguson KK et al, 2014	United States
Ferguson KK et al, 2015	United States
Ferguson KK et al, 2017	United States
Finkelstein JL et al, 2015	United States
Fischer ST et al, 2017	United States
Gao H et al, 2017	China
Gardner RM et al, 2011	Bangladesh
Ghartey J et al, 2017	United States
Graça G et al, 2010	Portugal

For peer review only

Graça G et al, 2012	Portugal	
Graça G et al, 2012 (b)	Portugal	
Hogeveen M et al, 2010	Netherlands	
Huang J et al, 2017	China	
Kalhan SC et al, 2003	United States	
Khalil AA et al, 2013	United Kingdom	
Kuc S et al, 2014	Netherlands	
Lenters V et al, 2013	Greenland, Poland, Ukraine	
Lenters V et al, 2016	Greenland, Poland, Ukraine	
Liu K et al, 2017	China	
Lopez-Espinosa MJ et al, 2015	Spain	
Marchlewicz EH et al, 2016	United States	
Minatoya M et al, 2017	Japan	
Minatoya M et al, 2017 (b)	Japan	
Minatoya M et al, 2018	Japan	
Murphy MM et al, 2007	Spain	There is no data about any pregnancy outcomes.

For peer review only

Odibo AO et al, 2011	United States	
Pinney SE et al, 2017	United States	
Polanska K et al, 2014	Poland	
Polanska K et al, 2014 (b)	Poland	
Porter A et al, 2018	United States	
Rejc B et al, 2016	Slovenia	
Rijvers CAH et al, 2013	Netherlands	
Robledo C et al, 2013	United States	
Sachse D et al, 2012	Norway	
Scholtens DM et al, 2016	United Kingdom	
Shisler S et al, 2017	United States	Not all analysis were performed with metabolomics approach.
Tamblyn JA et al, 2018	Ireland	Duplicate data. Check Kiely ME et al, 2016.
Thomas MM et al, 2015	New Zealand	
Van Lee L et al, 2015	Singapore	
Virgiliou C et al, 2017	Greece	
Walsh J et al, 2012	Ireland	

For peer review only

Wang PW et al, 2015	Taiwan	
Watkins DJ et al, 2016	United States	
Wolff MS et al, 2008	United States	
Woods MM et al, 2017	United States	
Yang P et al, 2018	China	
Duplicate data		
Horgan R et al, 2009	Australia	Check Horgan R et al, 2011.
Horgan R et al, 2011	Australia	Check Horgan R et al, 2011.
Khashan AS et al, 2013	Ireland	Check Kiely ME et al, 2016.
Sulek et al, 2014	Singapore	Check Sulek et al, 2014.

For peer review only

Examining the predictive accuracy of metabolomics for small for gestational age babies: a systematic review

Debora F. B. Leite & Aude-Claire Morillon, Elias F. Melo Júnior, Renato T. Souza, Fergus P. McCarthy, Ali S. Khashan, Philip N. Baker, Louise C. Kenny, José Guilherme Cecatti.

Supplementary material 3 - Individual QUADAS-2 data for all 15 included studies.

**Studies**	**Risk of bias**							
	**Patient selection**		**Index test**		**Reference standard**		**Flow and timing**	
	Was a consecutive or random sample of patients enrolled?	Did the study avoid inappropriate exclusions?	Were the index test results interpreted without knowledge of the results of the reference standard?	If a threshold was used, was it pre-specified?	Is the reference standard likely to correctly classify the target condition?	Were the reference standard results interpreted without knowledge of the results of the index test?	Did all patients receive the same reference standard?	Were all patients included in the analysis?
Grandone E et al, 2006	Yes	Yes	Unclear	No	No	Unclear	Yes	Yes
van Eijsden M et al, 2008	Yes	Yes	No	No	Yes	Yes	Yes	Yes
Horgan R et al, 2011	Yes	Yes	No	Yes	Yes	Yes	Yes	Yes
Costet N et al, 2012	Yes	Yes	Yes	No	Yes	Unclear	Yes	No
Ertl R et al, 2012	Yes	Yes	No	Yes	Yes	Yes	Yes	Yes
Gernand AD et al, 2013	Yes	Yes	No	Yes	Yes	Yes	Yes	Yes
Sulek K et al, 2014	Yes	Yes	No	Yes	Unclear	Unclear	Yes	Yes
Choi R et al, 2016	Yes	Yes	Unclear	Yes	Yes	Unclear	Yes	Yes
Kiely ME et al, 2016	Yes	Yes	No	Yes	Yes	Yes	Yes	Yes
Ong YL et al, 2016	Yes	Yes	No	Yes	Yes	Yes	Yes	No
Wang Y et al, 2016	Yes	Yes	No	No	Yes	Yes	Yes	Yes
Delplancke TDJ et al, 2018	Yes	Yes	No	Yes	Yes	Unclear	Yes	Yes
Luthra G et al, 2018	Yes	Yes	No	No	Yes	Yes	Yes	Yes
Gong S et al, 2018	No	Yes	No	No	Yes	Unclear	Yes	Yes
Morillon AC et al, 2018	Yes	Yes	No	Yes	Yes	Yes	Yes	Yes

For peer review only

Studies	Applicability concerns		
	Patient selection	Index test	Reference standard
	Are there concerns that the included patients do not match the review question?	Are there concerns that the index test, its conduct, or interpretation differ from the review question?	Are there concerns that the target condition as defined by the reference standard does not match the review question?
Grandone E et al, 2006	No	No	Yes
van Eijsden M et al, 2008	No	No	No
Horgan R et al, 2011	No	No	No
Costet N et al, 2012	No	No	No
Ertl R et al, 2012	No	No	No
Gernand AD et al, 2013	No	No	No
Sulek K et al, 2014	No	No	Yes
Choi R et al, 2016	Unclear	No	No
Kiely ME et al, 2016	No	No	No
Ong YL et al, 2016	No	No	No
Wang Y et al, 2016	No	No	No
Delplancke TDJ et al, 2018	No	Unclear	No
Luthra G et al, 2018	No	No	No
Gong S et al, 2018	Yes	Yes	No
Morillon AC et al, 2018	No	No	No

For peer review only

PRISMA 2009 Checklist

Examining the predictive accuracy of metabolomics for small for gestational age babies: a systematic review

Debora F. B. Leite & Aude-Claire Morillon, Elias F. Melo Júnior, Renato T. Souza, Fergus P. McCarthy, Ali S. Khashan, Philip N. Baker, Louise C. Kenny, José Guilherme Cecatti

Section/topic	#	Checklist item	Reported on page #
**TITLE**			
Title	1	Identify the report as a systematic review, meta-analysis, or both.	1
**ABSTRACT**			
Structured summary	2	Provide a structured summary including, as applicable: background; objectives; data sources; study eligibility criteria, participants, and interventions; study appraisal and synthesis methods; results; limitations; conclusions and implications of key findings; systematic review registration number.	3-4
**INTRODUCTION**			
Rationale	3	Describe the rationale for the review in the context of what is already known.	5
Objectives	4	Provide an explicit statement of questions being addressed with reference to participants, interventions, comparisons, outcomes, and study design (PICOS).	6
**METHODS**			
Protocol and registration	5	Indicate if a review protocol exists, if and where it can be accessed (e.g., Web address), and, if available, provide registration information including registration number.	6
Eligibility criteria	6	Specify study characteristics (e.g., PICOS, length of follow-up) and report characteristics (e.g., years considered, language, publication status) used as criteria for eligibility, giving rationale.	7-8
Information sources	7	Describe all information sources (e.g., databases with dates of coverage, contact with study authors to identify additional studies) in the search and date last searched.	6-7/ 9
Search	8	Present full electronic search strategy for at least one database, including any limits used, such that it could be repeated.	6-7
Study selection	9	State the process for selecting studies (i.e., screening, eligibility, included in systematic review, and, if applicable, included in the meta-analysis).	7-8
Data collection process	10	Describe method of data extraction from reports (e.g., piloted forms, independently, in duplicate) and any processes for obtaining and confirming data from investigators.	8
Data items	11	List and define all variables for which data were sought (e.g., PICOS, funding sources) and any assumptions and simplifications made.	7-8
Risk of bias in individual studies	12	Describe methods used for assessing risk of bias of individual studies (including specification of whether this was done at the study or outcome level), and how this information is to be used in any data synthesis.	8-9

For peer review only - http://bmjopen.bmj.com/site/about/guidelines.xhtml

PRISMA 2009 Checklist

Examining the predictive accuracy of metabolomics for small for gestational age babies: a systematic review

Debora F. B. Leite & Aude-Claire Morillon, Elias F. Melo Júnior, Renato T. Souza, Fergus P. McCarthy, Ali S. Khashan, Philip N. Baker, Louise C. Kenny, José Guilherme Cecatti

Summary measures	13	State the principal summary measures (e.g., risk ratio, difference in means).	9
Synthesis of results	14	Describe the methods of handling data and combining results of studies, if done, including measures of consistency (e.g., I^2) for each meta-analysis.	9

Section/topic	#	Checklist item	Reported on page #
Risk of bias across studies	15	Specify any assessment of risk of bias that may affect the cumulative evidence (e.g., publication bias, selective reporting within studies).	8-9
Additional analyses	16	Describe methods of additional analyses (e.g., sensitivity or subgroup analyses, meta-regression), if done, indicating which were pre-specified.	7
**RESULTS**			
Study selection	17	Give numbers of studies screened, assessed for eligibility, and included in the review, with reasons for exclusions at each stage, ideally with a flow diagram.	9
Study characteristics	18	For each study, present characteristics for which data were extracted (e.g., study size, PICOS, follow-up period) and provide the citations.	9-13
Risk of bias within studies	19	Present data on risk of bias of each study and, if available, any outcome level assessment (see item 12).	23-24
Results of individual studies	20	For all outcomes considered (benefits or harms), present, for each study: (a) simple summary data for each intervention group (b) effect estimates and confidence intervals, ideally with a forest plot.	20-22
Synthesis of results	21	Present results of each meta-analysis done, including confidence intervals and measures of consistency.	24
Risk of bias across studies	22	Present results of any assessment of risk of bias across studies (see Item 15).	9; 23-24
Additional analysis	23	Give results of additional analyses, if done (e.g., sensitivity or subgroup analyses, meta-regression [see Item 16]).	NA
**DISCUSSION**			
Summary of evidence	24	Summarize the main findings including the strength of evidence for each main outcome; consider their relevance to key groups (e.g., healthcare providers, users, and policy makers).	24-28
Limitations	25	Discuss limitations at study and outcome level (e.g., risk of bias), and at review-level (e.g., incomplete retrieval of identified research, reporting bias).	28-29
Conclusions	26	Provide a general interpretation of the results in the context of other evidence, and implications for future research.	29-30
**FUNDING**			

For peer review only

For peer review only - http://bmjopen.bmj.com/site/about/guidelines.xhtml

PRISMA 2009 Checklist

Examining the predictive accuracy of metabolomics for small for gestational age babies: a systematic review

Debora F. B. Leite & Aude-Claire Morillon, Elias F. Melo Júnior, Renato T. Souza, Fergus P. McCarthy, Ali S. Khashan, Philip N. Baker, Louise C. Kenny, José Guilherme Cecatti

Funding	27	Describe sources of funding for the systematic review and other support (e.g., supply of data); role of funders for the systematic review.	38-39

From: Moher D, Liberati A, Tetzlaff J, Altman DG, The PRISMA Group (2009). Preferred Reporting Items for Systematic Reviews and Meta-Analyses: The PRISMA Statement. PLoS Med 6(7): e1000097. doi:10.1371/journal.pmed1000097

For more information, visit: www.prisma-statement.org.

Page 2 of 2

For peer review only
